# Serum lipidomic analysis identifies potential therapeutic targets for neurodegeneration

**DOI:** 10.3389/fnhum.2025.1598495

**Published:** 2025-06-18

**Authors:** Lumi Zhang, Duanbin Li, Na Zhao, Guoping Peng

**Affiliations:** ^1^Department of Neurology, Wenzhou TCM Hospital of Zhejiang Chinese Medical University, Wenzhou, China; ^2^Department of Cardiology, Sir Run Run Shaw Hospital, School of Medicine, Zhejiang University, Hangzhou, China; ^3^Department of Neurology, The First Affiliated Hospital, Zhejiang University School of Medicine, Hangzhou, China

**Keywords:** VLSFA, NfL, neurodegeneration, hypertension, NHANES

## Abstract

**Introduction:**

Circulating very-long-chain saturated fatty acids (VLSFAs) may attenuate age-related cognitive decline, but their direct association with neurodegeneration biomarkers and the underlying mechanisms remain unclear.

**Methods:**

This cross-sectional study examined associations between circulating fatty acid profiles, neurodegeneration (assessed by serum neurofilament light chain, NfL), cognitive function, and hypertension in 1,677 U.S. adults from the National Health and Nutrition Examination Survey (NHANES) 2013–2014. Advanced statistical methods including weighted quantile sum (WQS) regression, Bayesian kernel machine regression (BKMR), quantile g-computation (Qgcomp), and formal mediation analyses were employed.

**Results:**

The VLSFA mixture demonstrated significant inverse associations with serum NfL (β = −0.044, 95% CI: -0.076, -0.011) and the prevalence of hypertension (OR = 0.788, 95% CI: 0.672, 0.923). This association with NfL was non-linear, exhibiting a more pronounced protective effect at lower VLSFA concentrations. Higher VLSFA levels were significantly correlated with better cognitive performance, particularly in processing speed (Digit-Symbol Substitution Test) and memory (delayed recall). Hypertension was positively associated with NfL (β=4.133, 95% CI: 1.705, 6.562), an effect driven primarily by systolic blood pressure. Mediation analysis revealed that hypertension accounted for approximately 15–20% of the total association between VLSFAs and NfL.

**Discussion:**

Circulating VLSFAs are inversely associated with the neurodegeneration biomarker NfL and positively correlated with cognitive performance. This neuroprotective association appears to be partially mediated by blood pressure regulation pathways. These findings identify VLSFAs as a potential therapeutic target, warranting further longitudinal and interventional studies to confirm their role in mitigating neurodegeneration.

## Highlights

- Serum lipidomic analysis identifies VLSFAs as inversely associated with NfL and positively with cognitive performance.
- Non-linear relationship suggests stronger neuroprotective associations at lower VLSFA levels.
- Hypertension mediates approximately 15–20% of the relationship between VLSFAs and neurodegeneration.
- Blood pressure regulation represents a potential mechanism linking VLSFAs to neurological health.

## 1 Introduction

As population aging accelerates, age-related neurodegenerative diseases present a growing challenge to healthcare systems worldwide (Yang et al., 2013). Although these diseases can be highly complex and etiologically diverse, neurodegeneration—a progressive process encompassing both structural damage to the nervous system, such as neuroaxonal injury, and ensuing functional decline, including cognitive impairment—stands as a common and pervasive pathological feature (Katsnelson et al., 2016). Key risk factors that contribute to neurodegeneration include chronic hypertension (Kruyer et al., 2015; Santisteban and Iadecola, 2018; Jeon et al., 2019), high inflammatory state (Glass et al., 2010), hypoxia (Peers et al., 2009), and lipid and immune abnormalities (Hallett et al., 2019). Among these, hypertension stands out due to its global prevalence and has been recognized as a major cause of age-related cognitive impairment (Iadecola and Gottesman, 2019). To easily monitor neurodegeneration, serum neurofilament light chain (NfL) has emerged as an instrumental biomarker. NfL is a component of the neuron-specific type IV intermediate filament and released after neuroaxonal damage. NfL shows high specificity and has been validated in several neurological diseases, including Parkinson’s disease (Aamodt et al., 2021), Alzheimer’s disease (Quiroz et al., 2020), multiple system atrophy (Chelban et al., 2022), and multiple sclerosis (Benkert et al., 2022). Consequently, NfL has become an essential tool for assessing the efficacy of interventions targeting neurodegeneration. Meanwhile, standardized cognitive function tests provide direct functional assessment of neurodegeneration, offering clinical relevance that complements molecular biomarkers (Gavett et al., 2018). Together, the measurement of NfL and cognitive assessments provide a comprehensive evaluation of neurodegenerative processes, combining molecular insights with functional outcomes.

Very-long-chain saturated fatty acids (VLSFAs), characterized by a carbon chain length greater than 20 without double bonds, hold potential as neuroprotective interventions in therapeutic applications (Lemaitre and King, 2022). Traditionally, the general public has been advised to reduce their intake of saturated fatty acids (FAs), as they are considered to be detrimental to health (Helgadottir et al., 2022). However, it is inappropriate to generalize the harm of all saturated FAs, as their health effects vary depending on the length of the carbon chain (Perna and Hewlings, 2022; Fretts et al., 2016). Extensive studies have demonstrated the cardiovascular benefits of VLSFAs in conditions such as hypertension (Li et al., 2020), heart failure (Lemaitre et al., 2018; Flock and Kris-Etherton, 2013), atrial fibrillation (Fretts et al., 2014), coronary artery disease (Malik et al., 2015; Papandreou et al., 2019), and sudden cardiac arrest (Lemaitre et al., 2014). Interestingly, the health benefits of VLSFAs may extend beyond the cardiovascular system. In the Atherosclerosis Risk in Communities (ARIC) cohort, researchers found that higher levels of circulating VLSFAs in midlife were associated with less cognitive decline 20 years later, suggesting a potential neuroprotective role for VLSFAs (Li et al., 2020). More recent cross-sectional analyses of National Health and Nutrition Examination Survey (NHANES) data from older adult populations have further explored these associations, albeit with some specificity for individual VLSFA species. For instance, higher circulating concentrations of docosanoic acid (C22:0) and lignoceric acid (C24:0) were found to be associated with better global cognitive function and delayed recall (Shen et al., 2024). Separately, higher serum tricosanoic acid (C23:0) levels have been linked to improved cognitive performance, with complementary evidence indicating reduced C23:0 expression in the frontal cortex of Alzheimer’s disease patients (Yang et al., 2025). Despite these emerging associations between specific circulating VLSFAs and cognitive outcomes, the underlying mechanisms and potential involvement of molecular pathways remain unclear. Further validation of the neurological benefits of VLSFAs and exploration of their mechanisms are warranted.

Therefore, building upon the longitudinal cognitive findings from the ARIC cohort which highlighted a gap in understanding the underlying mechanisms, this cross-sectional study aims to advance the field by investigating the association between VLSFAs (both independently and jointly) and neurodegeneration within the NHANES 2013–2014 dataset. Specifically, we extend prior work by: (1) Utilizing serum NfL, a sensitive molecular biomarker of neuroaxonal damage, in conjunction with functional cognitive performance measures to provide a multi-faceted assessment of neurodegeneration, thereby complementing previous cognitive-only studies. (2) Explicitly exploring the potential mediating role of hypertension, a key modifiable cardiovascular risk factor linked to both VLSFAs and neurodegeneration, to elucidate potential biological pathways underlying the observed associations. (3) Employing sophisticated statistical mixture models (WQS, BKMR, Qgcomp) to assess the combined impact of VLSFAs, offering insights beyond single-lipid analyses.

## 2 Methods

### 2.1 Study population

This study utilized data from the 2013–2014 cycle of the National Health and Nutrition Examination Survey (NHANES), which included measurements of both circulating FA profiles and serum NfL. NHANES assesses the health and nutritional status of the U. S. population through various data collection methods (Johnson et al., 2013).

From the initial 10,175 participants, we included those with circulating FA profile measurements (*N* = 3,987) and excluded those with missing serum NfL data (*N* = 2,122), missing hypertension diagnosis data (*N* = 54), estimated glomerular filtration rate (eGFR), calculated using the Chronic Kidney Disease Epidemiology Collaboration (CKD-EPI) equation, <60 mL/min/1.73m^2^ (*N* = 91), and history of brain cancer or stroke (*n* = 43). The final study population comprised 1,677 community-dwelling general US individuals (Supplementary Figure S1).

### 2.2 Circulating fatty acid profiles

During the processing of circulating FA profiles, esterified FAs were first hydrolyzed from triglycerides, phospholipids, and cholesterol esters. This sequential process involved treatment with mineral acids and bases under heated conditions. Total FAs were then extracted from the matrix (100 μL fasting serum or plasma) using hexane, followed by derivatization and separation. Selected ion monitoring and electron capture negative-ion mass spectrometry were used to resolve individual FAs of interest. Finally, the profiles of the 30 FAs were quantified based on peak area and corrected with internal standards. For a more complete understanding of the FAs assessment methodology, please refer to the laboratory method files (CDC, 2014a).

The lower limit of detection (LLOD) values for the FA profiles are presented in Supplementary Table S1 and show nearly 100% detection rates for all, except for capric acid (C10:0), which has a detection rate of 55.5%. The circulating FAs (30 kinds) were further categorized into distinct subtypes based on the number of double bonds (0: saturated; 1: monounsaturated; >1: polyunsaturated) and carbon chain lengths (6–11: medium chain; 12–20: long chain; >20: very long chain). Among these, we focused primarily on the subtype of VLSFAs, which were characterized by carbon chain lengths greater than 20 and no double bonds, including docosanoic acid (C22:0), tricosanoic acid (C23:0), and lignoceric acid (C24:0).

### 2.3 Neurodegeneration and serum NfL

Serum NfL, a specific biomarker of neurodegeneration, was quantified using a highly sensitive immunoassay developed by Siemens Healthineers. The assay employs acridinium ester chemiluminescence and paramagnetic particles on the Attelica platform—an existing automated high-throughput system. Procedural steps include sample incubation, addition of capture antibody coated paramagnetic particles, separation, and the initiation of automated chemiluminescence. For a complete understanding of the serum NfL assessment, please refer to the laboratory method files (CDC, 2014b).

### 2.4 Hypertension and mediation assumption

Blood pressure (BP) measurements were performed in the mobile examination center by certified examiners following a standardized procedure. Participants rested quietly in a seated position for 5 min before at least three consecutive BP measurements were taken. Hypertension was defined as systolic blood pressure (SBP) ≥ 140 mmHg, diastolic blood pressure (DBP) ≥ 90 mmHg, or use of antihypertensive medication.

The hypothesis that hypertension may mediate the relationship between VLSFAs and neurodegeneration is supported by previous studies showing associations between VLSFAs and cognitive decline (Li et al., 2020), VLSFAs and hypertension risk (Li et al., 2020), and chronic hypertension and age-related cognitive impairment (Iadecola and Gottesman, 2019). Specifically, hypertension was selected as the primary candidate mediator for this study due to this existing evidence: (a) prior research indicates an inverse association between circulating VLSFAs and hypertension risk or blood pressure levels (Li et al., 2020), and (b) chronic hypertension is a well-established and highly prevalent risk factor for neurodegeneration and age-related cognitive impairment (Kruyer et al., 2015; Santisteban and Iadecola, 2018; Jeon et al., 2019; Iadecola and Gottesman, 2019). These established links render hypertension a plausible and clinically relevant pathway through which VLSFAs might exert neuroprotective effects.

### 2.5 Covariates

Participant characteristics were extracted from NHANES (2013–2014) to define covariates. Alcohol consumption and smoking status were categorized based on frequency and history. Leisure-time physical activity (PA) was categorized as none, moderate, or vigorous. The family poverty income ratio (PIR) was computed as family income divided by poverty guidelines. Body mass index (BMI, kg/m^2^) was calculated as weight (kilograms) divided by height (meters) squared. Diet quality was assessed using the Healthy Eating Index-2015 (HEI-2015) (Krebs-Smith et al., 2018), with detailed components and scoring standards provided in Supplementary Table S2. HEI-2015 was included as a covariate to account for overall dietary patterns, distinct from specific circulating VLSFA levels. While some dietary components influencing HEI-2015 scores may correlate with VLSFA intake (e.g., nuts, seeds), VLSFAs constitute a minor fraction of total dietary fat considered by this index. Adjusting for HEI-2015 allows for a more robust assessment of the independent associations of circulating VLSFAs, given that these are influenced by both dietary intake and complex endogenous metabolic pathways.

### 2.6 Statistical analysis

Statistical analyses were conducted using R software (version 4.4.1). Normally distributed continuous variables were expressed as mean ± standard deviation and compared using an independent t-test. Non-normally distributed continuous variables were presented as median [interquartile range] and compared using the Mann–Whitney U test. Categorical variables were reported as counts (percentages) and analyzed with the chi-square test. The concentration distribution of circulating fatty acid (FA) profiles (30 types) was visualized using a ridge plot. Pairwise correlations of Ln-transformed FAs were assessed using Pearson correlation analysis.

Linear regression models examined associations between FAs and NfL, as well as hypertension and NfL. To minimize confounding, trends between VLSFAs and NfL were visually depicted using a locally weighted scatterplot smoothing (Lowess) curve (span = 0.9). Logistic regression models examined associations between FAs and hypertension. Participants were equally divided into four groups (Q1 to Q4) according to VLSFA concentrations. A trend test for coefficients across VLSFA groups was performed by entering the median value of VLSFAs for each group as a numerical variable. This association was further visualized using a restricted cubic spline (RCS) model with four knots at the 5th, 35th, 65th, and 95th percentiles of each VLSFA. Subgroup analyses were performed between VLSFAs and NfL, as well as VLSFAs and hypertension, stratified by age, sex, BMI, triglyceride, fasting blood glucose (FBG), HEI-2015 score, and leisure-time physical activity (PA).

To examine joint associations of FA subtypes with NfL and hypertension, three co-exposure models were employed: weighted quantile sum (WQS) regression (Carrico et al., 2015), Bayesian kernel machine regression (BKMR) (Bobb et al., 2015) and Quantile g-computation (Qgcomp) (Keil et al., 2020). The WQS index reflects the contribution of each FA to the joint exposure, ranging from 0 to 1. BKMR is a semi-parametric method allowing for exploration of nonlinear and nonadditive relationships, estimating effects of the overall mixture, individual components, and interactions. Qgcomp integrates the flexibility of g-computation, inference simplicity of WQS, and non-additivity and non-linearity of BKMR. Traditional regression methods face challenges when analyzing highly correlated exposures like circulating fatty acids (FAs), as observed in our data (Supplementary Figure S3). Analyzing FAs individually ignores their combined action and increases risks of false positives, while including correlated FAs in standard multivariable models can cause multicollinearity issues (e.g., unstable estimates). Furthermore, these methods do not directly estimate the overall effect of the FA mixture. Therefore, we employed advanced mixture modeling techniques. WQS regression estimates a weighted index representing the joint effect of the VLSFA mixture and identifies key contributors. BKMR offers a flexible non-parametric approach to model complex non-linear relationships and interactions within the mixture. Qgcomp provides a complementary parametric method to estimate the overall mixture effect. Using these three distinct approaches enhances the robustness of our findings regarding the joint associations of VLSFAs.

For BKMR, a Gaussian kernel was used for the continuous outcome NfL, and a probit link function for binary outcome hypertension. BKMR models were fitted with 10,000 iterations using the Markov Chain Monte Carlo sampler. The conditional posterior inclusion probability was calculated to indicate the importance of each VLSFA, with a probability greater than 0.5 indicating relative importance. For Qgcomp, the q parameter was fixed at 4, with confidence intervals determined by 500 bootstrap iterations.

A parallel mediation model assessed the proportion of mediating effect, with hypertension as the underlying mediator. The quasi-Bayesian Monte Carlo method was employed in the mediation analysis with 200 simulations and normal approximation.

Sensitivity analyses included: (1) reassessing the association between VLSFAs and hypertension in an expanded population (*N* = 3,192); (2) estimating correlations between dietary FA intakes and circulating concentrations; (3) reassessing the association between circulating VLSFAs and NfL after adjusting for dietary FA intake; (4) assessing the association between serum NfL concentrations and cognitive function in a subset population (*N* = 418); and (5) assessing the association between circulating VLSFAs and cognitive function in this subset population.

All models were adjusted for covariates including age, sex, race/ethnicity, BMI, educational attainment, poverty income ratio, marital status, smoking status, alcohol consumption, leisure-time PA, HEI-2015 score, FBG, triglyceride, and serum creatinine.

## 3 Results

### 3.1 Population characteristics

In Table 1, we summarized the characteristics of 1,677 US community-dwelling general individuals (mean age, 47.1 years; male, 47.4%) based on the median stratification of serum neurofilament light chain (NfL) concentration (median value = 12.4 pg./mL). Comparison with the low NfL group, participants in the high NfL group were older (54.1 ± 14.4 vs. 39.9 ± 12.9 years, *p* < 0.001), more likely to be male (51.1 vs. 43.7%, *p* = 0.003), and had higher serum creatinine levels (0.88 [0.74, 1.04] vs. 0.80 [0.69, 0.93] mg/dL, *p* < 0.001), and an increased prevalence of hypertension (48.5 vs. 24.3%, *p* < 0.001), especially with an elevated systolic blood pressure (SBP) (125.2 ± 19.1 vs. 118.0 ± 15.3 mmHg, *p* < 0.001). In addition, the concentration of most fatty acids (FAs) was elevated in the high NfL group (Supplementary Table S3).

**Table 1:** Baseline characteristics.

Characteristics	Total	Median of serum NfL		*P* value
		<12.4 pg/mL	≥12.4 pg/mL	
*N*	1,677	836	841	
Serum NfL, pg./mL	12.4 [8.4, 19.3]	8.4 [6.3, 10.1]	19.3 [15.2, 26.9]	
Age, years	47.06 ± 15.40	39.94 ± 12.90	54.13 ± 14.38	<0.001
Male (%)	795 (47.4)	365 (43.7)	430 (51.1)	0.003
Race/ethnicity (%)				<0.001
Non-Hispanic White	698 (41.6)	300 (35.9)	398 (47.3)	
Non-Hispanic Black	319 (19.0)	175 (20.9)	144 (17.1)	
Mexican American	234 (14.0)	141 (16.9)	93 (11.1)	
Other Hispanic	177 (10.6)	87 (10.4)	90 (10.7)	
Other Race	249 (14.8)	133 (15.9)	116 (13.8)	
BMI, kg/m^2^	29.31 ± 7.51	29.51 ± 7.72	29.11 ± 7.29	0.275
Marital status (%)				0.363
Married or living with partner	1,036 (61.8)	526 (62.9)	510 (60.6)	
others	641 (38.2)	310 (37.1)	331 (39.4)	
Poverty income ratio (%)				0.220
<1	397 (23.7)	213 (25.5)	184 (21.9)	
1–3	617 (36.8)	299 (35.8)	318 (37.8)	
>3	663 (39.5)	324 (38.8)	339 (40.3)	
Educational attainment (%)				0.601
Less than high school	120 (7.2)	55 (6.6)	65 (7.7)	
High school or equivalent	578 (34.5)	294 (35.2)	284 (33.8)	
College or above	979 (58.4)	487 (58.3)	492 (58.5)	
Smoking status (%)				<0.001
Never	959 (57.2)	525 (62.8)	434 (51.6)	
Former	376 (22.4)	149 (17.8)	227 (27.0)	
Current	342 (20.4)	162 (19.4)	180 (21.4)	
Alcohol consumption (%)				<0.001
Never	308 (18.4)	161 (19.3)	147 (17.5)	
None in the past year	235 (14.0)	88 (10.5)	147 (17.5)	
Less 1 drink/month	331 (19.7)	163 (19.5)	168 (20.0)	
1 to 3 drinks/month	277 (16.5)	171 (20.5)	106 (12.6)	
1 to 3 drinks/week	374 (22.3)	192 (23.0)	182 (21.6)	
>4 drinks/week	152 (9.1)	61 (7.3)	91 (10.8)	
Leisure-time PA (%)				0.001
No or unable	832 (49.6)	395 (47.2)	437 (52.0)	
Moderate	439 (26.2)	206 (24.6)	233 (27.7)	
Vigorous	406 (24.2)	235 (28.1)	171 (20.3)	
HEI-2015 score	51.53 ± 15.15	51.05 ± 15.23	52.01 ± 15.06	0.196
Hypertension (%)	611 (36.4)	203 (24.3)	408 (48.5)	<0.001
SBP, mmHg	121.62 ± 17.64	118.04 ± 15.28	125.18 ± 19.05	<0.001
DBP, mmHg	69.22 ± 11.33	68.93 ± 10.71	69.51 ± 11.91	0.298
FBG, mmol/L	5.75 ± 1.95	5.39 ± 1.10	6.10 ± 2.47	<0.001
Serum creatinine, mg/dL	0.84 [0.70, 0.99]	0.80 [0.69, 0.93]	0.88 [0.74, 1.04]	<0.001
Triglyceride, mmol/L	1.06 [0.73, 1.61]	0.98 [0.66, 1.45]	1.17 [0.82, 1.71]	<0.001

Continuous variables with normal distribution were expressed as mean ± standard deviation and compared using an independent *t*-test. Continuous variables with non-normal distribution were expressed as median [interquartile range] and compared using the Mann–Whitney U test. Categorical variables were expressed as counts (percentages), and compared using the chi-square test. NfL indicates neurofilament light chain; BMI, body mass index; PA, physical activity; HEI-2015, healthy eating index-2015; SBP, systolic blood pressure; DBP, diastolic blood pressure; FBG, fasting blood glucose.

The concentration distribution of the FA profiles was remarkably different, with median concentrations of very-long-chain saturated fatty acids (VLSFAs) being 66 μmol/L for docosanoic acid (C22:0), 28.1 μmol/L for trisanoic acid (C23:0), and 56 μmol/L for lignocellulosic acid (C24:0) (Supplementary Figure S2). In correlation analyses, FAs with similar structures (e.g., similar chain lengths and double bonds) showed more pronounced positive correlations (Supplementary Figure S3).

### 3.2 FA profiles and NfL

**Figure 1:**
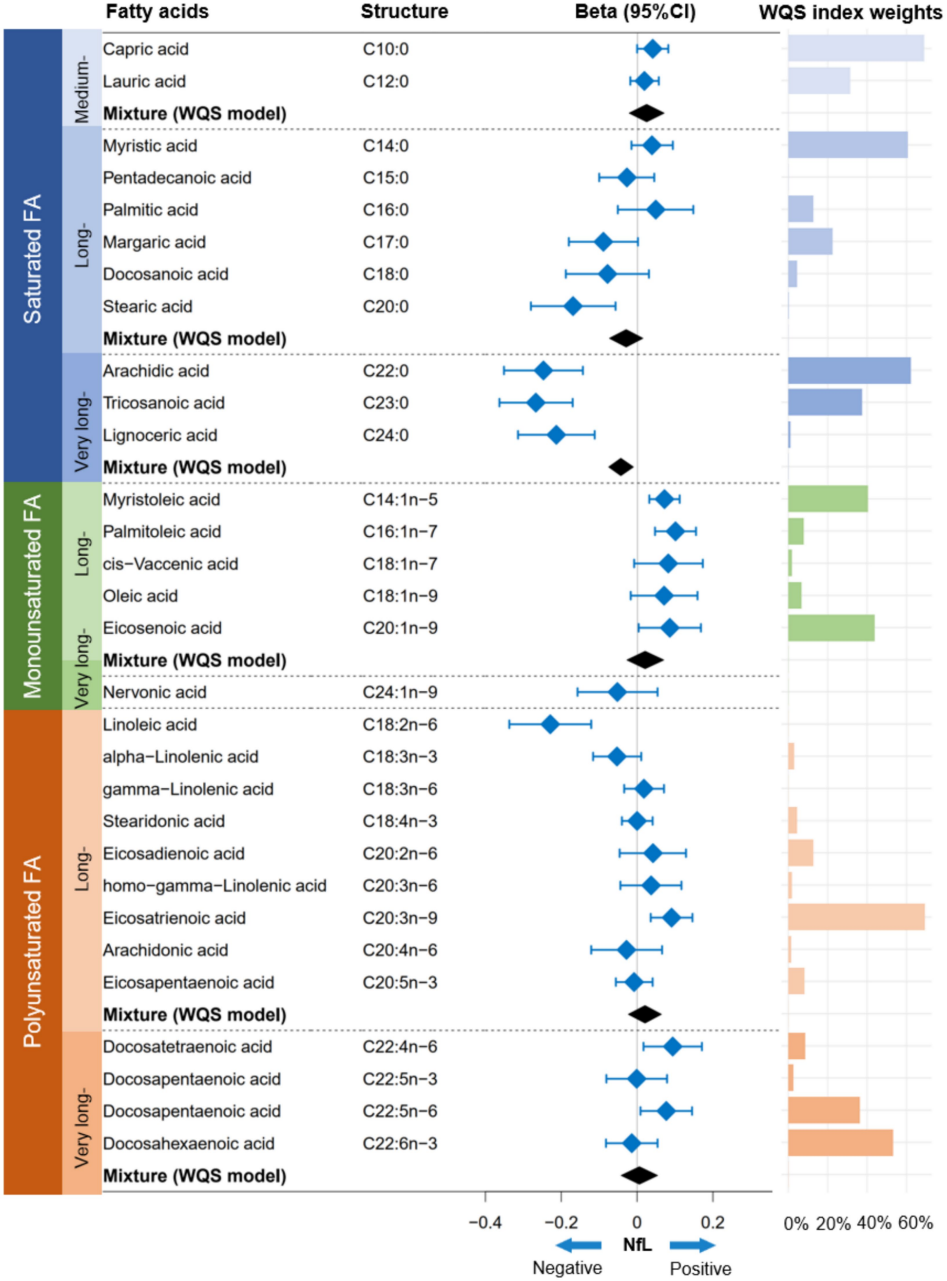
Association between FA profiles and serum NfL concentrations. The linear regression model and WQS model were employed to estimate the independent and joint associations between FA profiles and NfL, respectively. Prior to analysis, the concentrations of FA profiles and NfL were natural log-transformed to achieve a normal distribution. The models were adjusted for covariates, including age, sex, race/ethnicity, BMI, educational attainment, poverty income ratio, marital status, smoking status, alcohol consumption, leisure-time physical activity, Healthy Eating Index-2015, fasting blood glucose, triglyceride, and serum creatinine. NfL indicates neurofilament light chain; FA, fatty acid; WQS, weighted quantile sum; CI, confidence interval.

**Figure 2:**
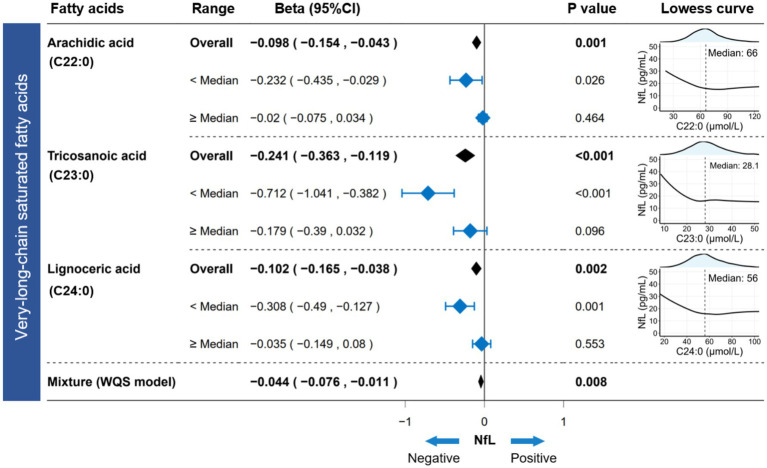
Association between circulating VLSFAs and serum NfL. The Lowess curve (span = 0.9) clarified the underlying trends between VLSFAs and NfL. The linear regression model estimated the independent association between VLSFAs and NfL in different ranges (overall/<median/≥median). WQS models provided parametric estimation of the joint association between VLSFAs and NfL. The models were adjusted for covariates, including age, sex, race/ethnicity, BMI, educational attainment, poverty income ratio, marital status, smoking status, alcohol consumption, leisure-time physical activity, Healthy Eating Index-2015, fasting blood glucose, triglyceride, and serum creatinine. NfL indicates neurofilament light chain; VLSFA, very-long-chain saturated fatty acid; Lowess, locally weighted scatterplot smoothing; WQS, weighted quantile sum; CI, confidence interval.

In the FA profiles, a negative association with serum NfL concentrations was found for several FAs, namely C20:0, C22:0, C23:0, C24:0, and C18:2n-6 (Figure 1). Regarding the joint association, only the VLSFA subtype was negatively associated with serum NfL (weighted quantile sum [WQS] model: Beta [95%CI] = −0.044 [−0.076, −0.011], *p* value = 0.008) (Figures 1, 2). In the WQS model, the index weights indicated that C22:0 and C23:0 contribute approximately 60 and 40%, respectively, highlighting their significant role in the association between VLSFAs and NfL (Figure 1).

### 3.3 VLSFAs and NfL

The Lowess curve showed a distinct downward trend of NfL as VLSFAs increased, especially when VLSFA levels were relatively low (Figure 2). This suggested that VLSFAs may be an essential component for maintaining the integrity of neural structures. For participants with VLSFA concentrations below the median level, each unit (1 μmol/L) increase in VLSFA was associated with a significant decrease in serum NfL—approximately 0.232 pg./mL for C22:0, 0.712 pg./mL for C23:0, and 0.308 pg./mL for C24:0 (Figure 2).

**Figure 3:**
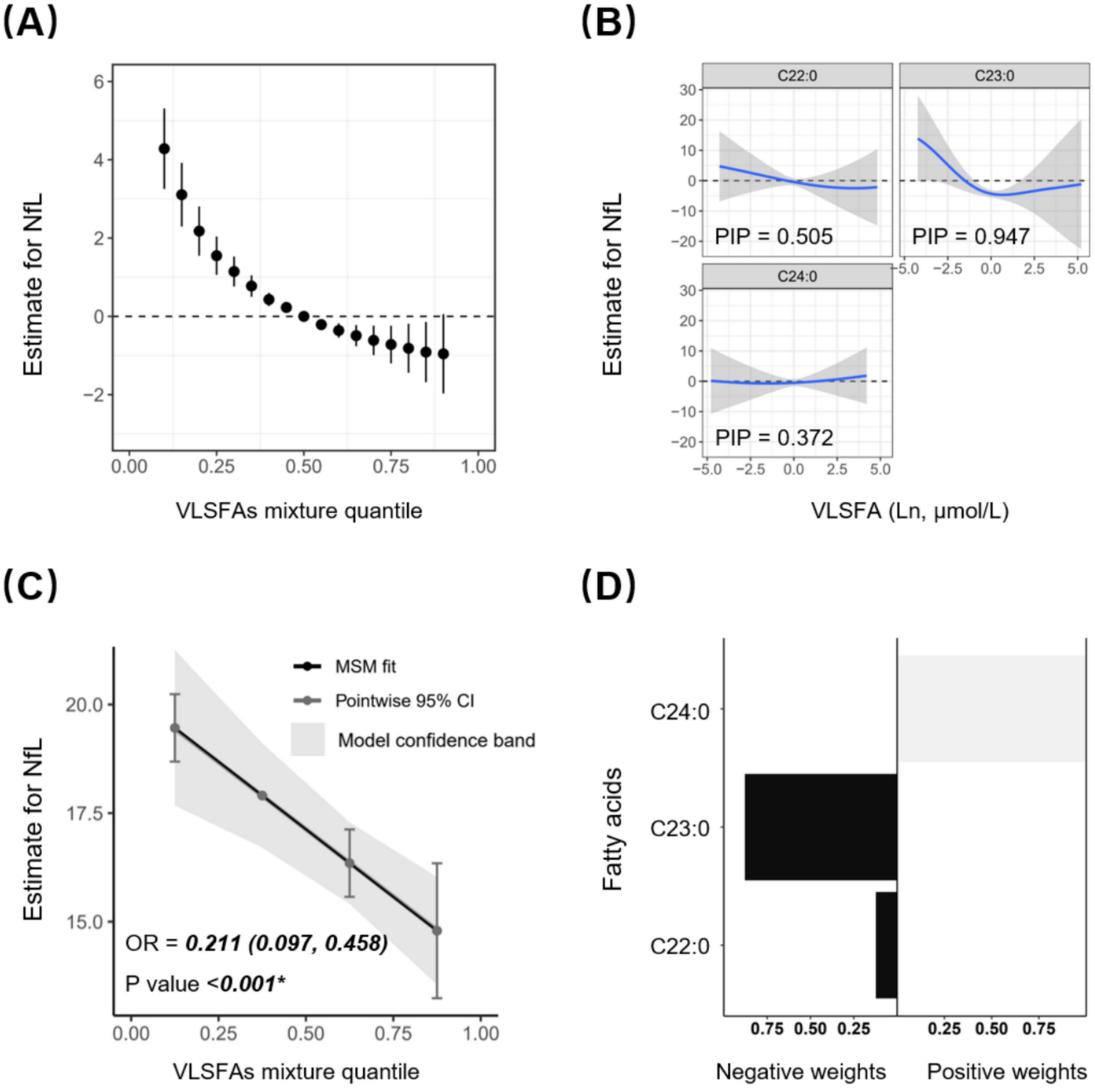
Joint association between circulating VLSFAs and serum NfL by BKMR and Qgcomp models. The BKMR model shows the joint estimates with 95% CI between VLSFAs and NfL **(A)**, as well as the dose–response association between specific VLSFA and NfL when the remaining VLSFA concentrations are fixed **(B)**. All VLSFAs at certain percentiles (increasing by 0.05) were compared to VLSFAs at the 50th percentile. Posterior Inclusion Probability (PIP) acts as an indicator of the relative importance of each variable in the BKMR model, with values closer to 1 indicating greater significance. The Qgcomp model presents the joint estimates with precise parametric calculation between VLSFAs and NfL **(C)**, along with the positive or negative regression weights for specific VLSFAs **(D)**. The models were adjusted for covariates, including age, sex, race/ethnicity, BMI, educational attainment, poverty income ratio, marital status, smoking status, alcohol consumption, leisure-time physical activity, Healthy Eating Index-2015, fasting blood glucose, triglyceride, and serum creatinine. NfL indicates neurofilament light chain; VLSFA, very-long-chain saturated fatty acid; BKMR, Bayesian kernel machine regression; Qgcomp, Quantile g-computation.

The Bayesian kernel machine regression (BKMR) and Quantile g-computation (Qgcomp) models further verified the robustness of the joint association between VLSFAs and NfL. The BKMR model confirmed that this negative association was more pronounced when VLSFAs were relatively low (Figure 3A) and was mainly contributed by C23:0 with a posterior inclusion probability of 0.947 (Figure 3B). Furthermore, the Qgcomp model quantified the negative joint association (OR = 0.211 [0.097, 0.458], *p* < 0.001) (Figure 3C) and showed that the negative weights were mainly contributed by C22:0 and C23:0 (Figure 3D).

**Figure 4:**
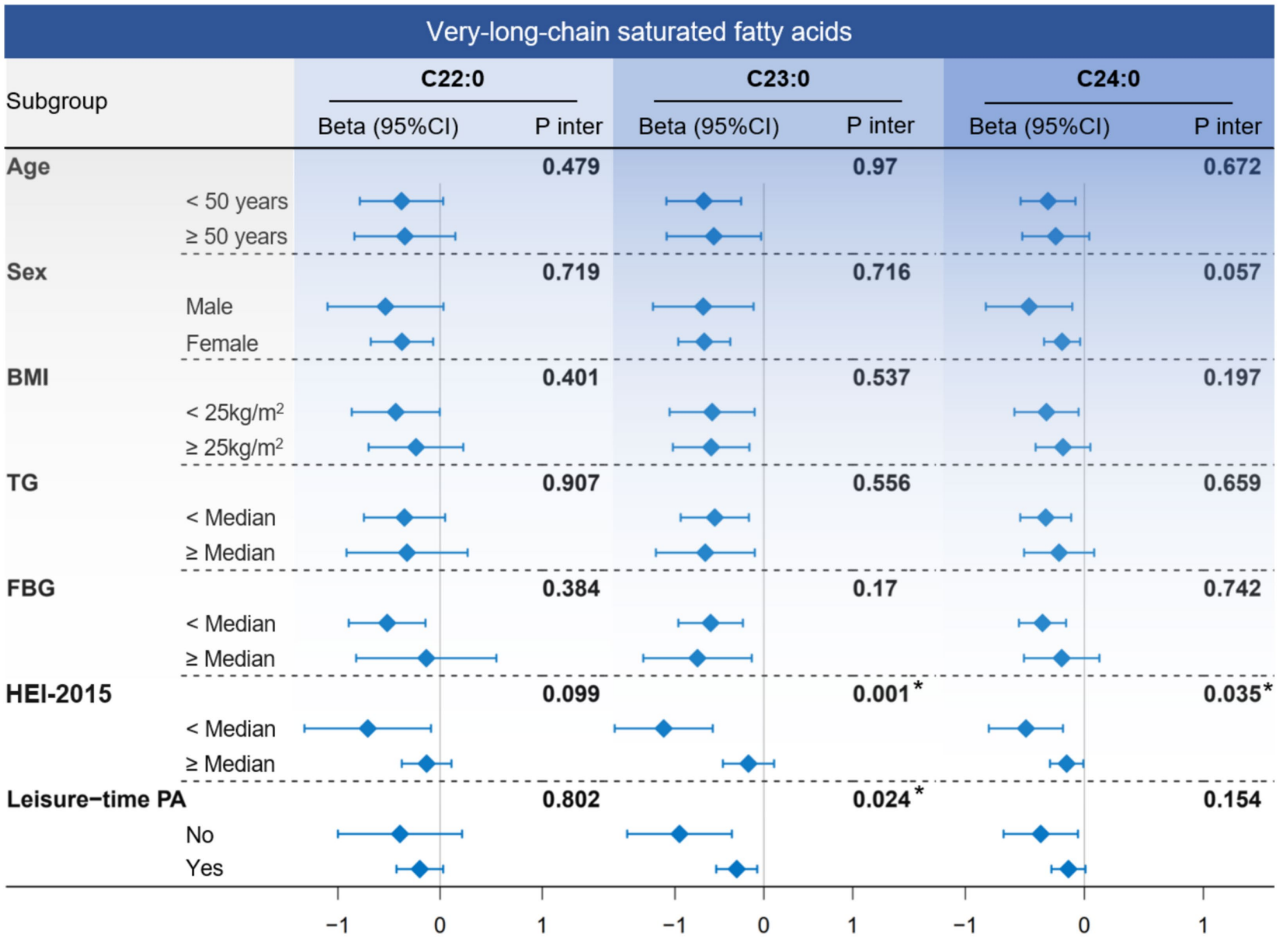
The subgroup analysis between circulating VLSFAs and serum NfL. The association between VLSFAs and NfL was estimated using a linear regression model when stratified by age, sex, BMI, TG, FBG, HEI-2015, and leisure-time PA. The median values of TG, FBG, and HEI-2015 were 1.06 mmol/L, 5.27 mmol/L, and 50.8, respectively. The models were adjusted for covariates, including age, sex, race/ethnicity, BMI, educational attainment, poverty income ratio, marital status, smoking status, alcohol consumption, leisure-time PA, HEI-2015, FBG, triglyceride, and serum creatinine (except for the stratified variable itself). NfL indicates neurofilament light chain; VLSFA, very-long-chain saturated fatty acid; FBG, fasting blood glucose; TG, triglyceride; HEI-2015, Healthy Eating Index-2015; PA, physical activity.

In subgroup analyses (Figure 4), the negative association between VLSFA and NfL remained consistent across the stratified population. Notably, participants with a low Healthy Eating Index-2015 (HEI-2015) score (poor diet quality) were more likely to benefit from increased levels of C23:0 (P for interaction = 0.001) and C24:0 (P for interaction = 0.035), suggesting that dietary behavior played a critical role in the association between VLSFAs and NfL.

### 3.4 VLSFAs and cognitive function

To validate the neuroprotective effects of VLSFAs observed in relation to NfL, we further examined the association between VLSFAs and cognitive function in a subset of participants who underwent cognitive assessment (*N* = 418). The cognitive evaluation included the CERAD Word Learning Subtest (immediate and delayed recall), the Animal Fluency Test, and the Digit Symbol Substitution Test (DSST).

Higher circulating VLSFA concentrations were significantly associated with better cognitive performance (Supplementary Table S4), particularly in DSST scores (C22:0: β = 7.952 [2.298, 13.606]; C23:0: β = 8.809 [3.311, 14.306]; C24:0: β = 7.203 [1.763, 12.643]; all *p* < 0.05) and delayed recall performance (C22:0: β = 0.827 [0.020, 1.635]; C23:0: β = 0.841 [0.055, 1.628]; all *p* < 0.05). These findings align with the inverse association between VLSFAs and NfL identified in the primary analysis (Supplementary Table S5), further supporting the potential neuroprotective role of VLSFAs.

### 3.5 FA profiles and hypertension

**Figure 5:**
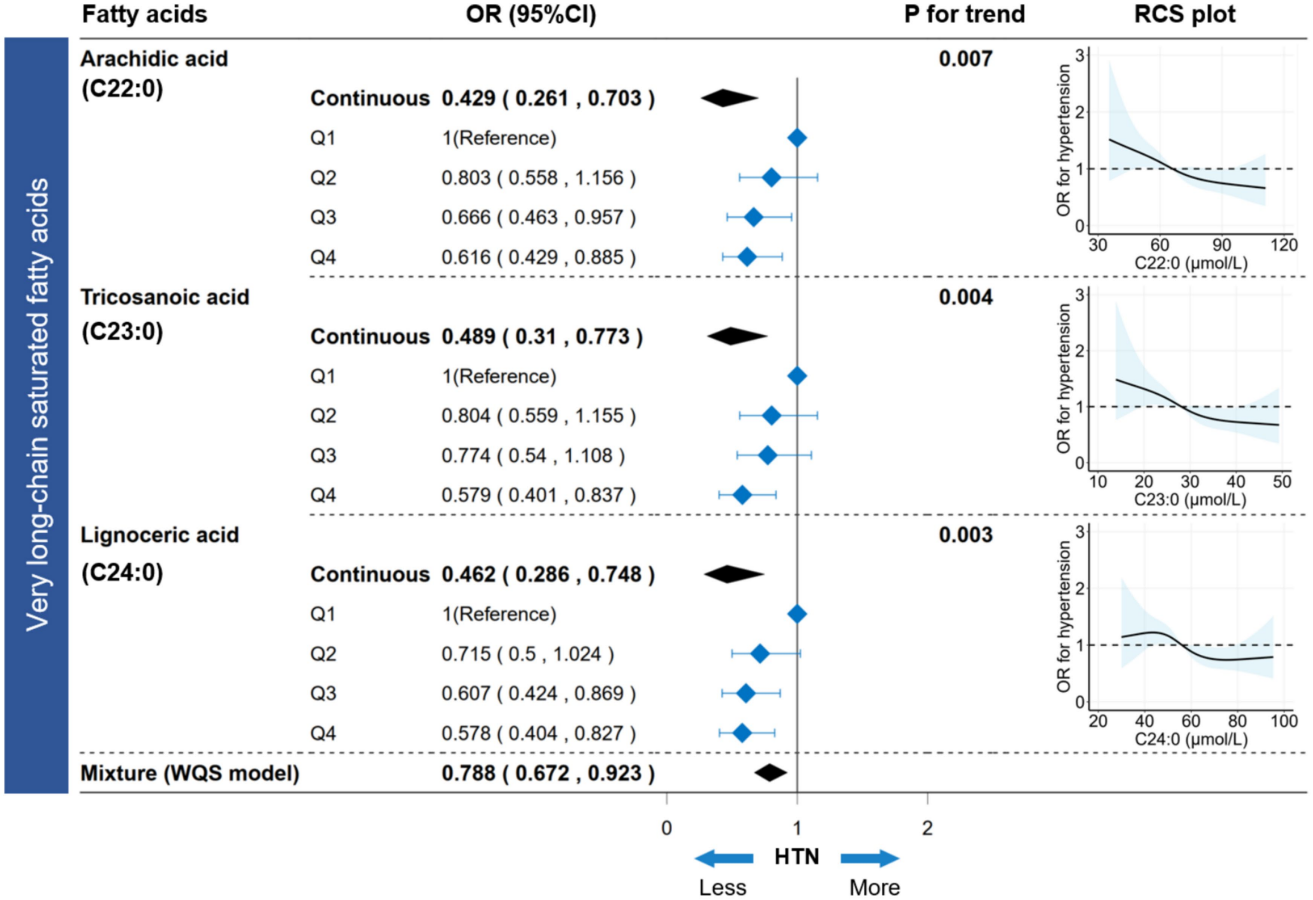
Association between circulating VLSFAs and hypertension. The association between VLSFAs (continuous or categorical) and hypertension was estimated using a logistic regression model. WQS models provided parametric estimation of the joint association between VLSFAs and hypertension. The restricted cubic spline (RCS) model visualized the dose–response association between VLSFAs and hypertension. A trend test for coefficients across VLSFA categories was performed using the median VLSFA concentration for each category as a numeric variable. The models were adjusted for covariates, including age, sex, race/ethnicity, BMI, educational attainment, poverty income ratio, marital status, smoking status, alcohol consumption, leisure-time physical activity, Healthy Eating Index-2015, fasting blood glucose, triglyceride, and serum creatinine. VLSFA indicates very-long-chain saturated fatty acid; WQS, weighted quantile sum; OR, odds ratio; RCS, restricted cubic spline.

In the FA profiles, a negative association with hypertension was found for several FAs, namely C20:0, C22:0, C23:0, C24:0, and C24:1n-9 (Supplementary Figure S4). Regarding the joint association, only the VLSFA subtype was negatively associated with hypertension (WQS model: OR [95%CI] = 0.788 [0.672, 0.923], *p* value < 0.001) (Supplementary Figure S4; Figure 5). In the WQS model, the index weights indicated that C23:0 contributed approximately 80%, highlighting its significant role in the association between VLSFAs and hypertension (Supplementary Figure S4).

### 3.6 VLSFAs and hypertension

The participants were equally divided into four groups (Q1-Q4) based on concentrations increment of VLSFAs (Supplementary Table S6). Compared to the Q1 group, the Q4 group showed a decreased risk of hypertension for C22:0 (OR [95% CI] = 0.616 [0.429, 0.885]), C23:0 (OR [95% CI] = 0.579 [0.401, 0.837]), and C24:0 (OR [95% CI] = 0.578 [0.404, 0.827]), with all *p* values for trend < 0.05 (Figure 5). Additionally, the dose–response RCS plot showed a gradual decrease in the risk of hypertension with increasing VLSFA concentrations (Figure 5). Moreover, the BKMR and Qgcomp models verified the robustness of the joint association between VLSFAs and NfL (Supplementary Figure S5). In subgroup analyses (Supplementary Figure S6), the inverse association between VLSFAs and hypertension remained consistent across the stratified population. Notably, the elderly (age ≥ 50 years) were more likely to benefit from increased VLSFAs, including C22:0 (P for interaction = 0.001), C23:0 (P for interaction = 0.046), and C24:0 (P for interaction = 0.027).

### 3.7 Hypertension and NfL

In Table 2, hypertension showed a positive association with serum NfL concentrations (Beta [95% CI] = 4.133 [1.705, 6.562]). Compared with diastolic blood pressure (DBP), the positive association between SBP and NfL was more pronounced, suggesting that SBP has a greater impact on neurodegeneration. This finding remained robust when NfL was further subjected to a natural logarithmic transformation.

**Table 2:** Association between blood pressure status and serum NfL concentrations.

Blood pressure status	Serum NfL (pg/mL)			Serum NfL (pg/mL, Ln-transformed)		
	Beta	95% CI	*P* value	Beta	95% CI	*P* value
HTN	4.133	(1.705, 6.562)	0.001*	0.083	(0.017, 0.148)	0.013*
Uncontrolled HTN	3.240	(0.327, 6.154)	0.029*	0.100	(0.022, 0.178)	0.012*
SBP, mmHg						
≥140	1.596	(−1.409, 4.601)	0.298	0.075	(−0.005, 0.155)	0.066
≥160	9.174	(3.461, 14.887)	0.002*	0.239	(0.086, 0.391)	0.002*
≥180	11.604	(1.067, 22.142)	0.031*	0.396	(0.115, 0.676)	0.006*
DBP, mmHg						
≥90	5.359	(0.034, 10.685)	0.049*	0.125	(−0.017, 0.267)	0.084
≥100	2.185	(−10.102, 14.473)	0.727	0.257	(−0.07, 0.584)	0.124
≥110	9.460	(−13.929, 32.850)	0.428	0.537	(−0.084, 1.159)	0.090

The association between blood pressure status and NfL (with or without Ln-transformed) was estimated by linear regression models. Models were adjusted for covariates, including age, sex, race/ethnicity, BMI, educational attainment, poverty income ratio, marital status, smoking status, alcohol consumption, leisure-time physical activity, Healthy Eating Index-2015, fasting blood glucose, triglyceride, serum creatinine. Uncontrolled HTN was defined as a diagnosis of hypertension with a measured SBP ≥ 140 mmHg or DBP ≥ 90 mmHg. NfL indicates neurofilament light chain; HTN, hypertension; SBP, systolic blood pressure; DBP, diastolic blood pressure. ^*^P value < 0.05.

### 3.8 Mediation analysis

**Figure 6:**
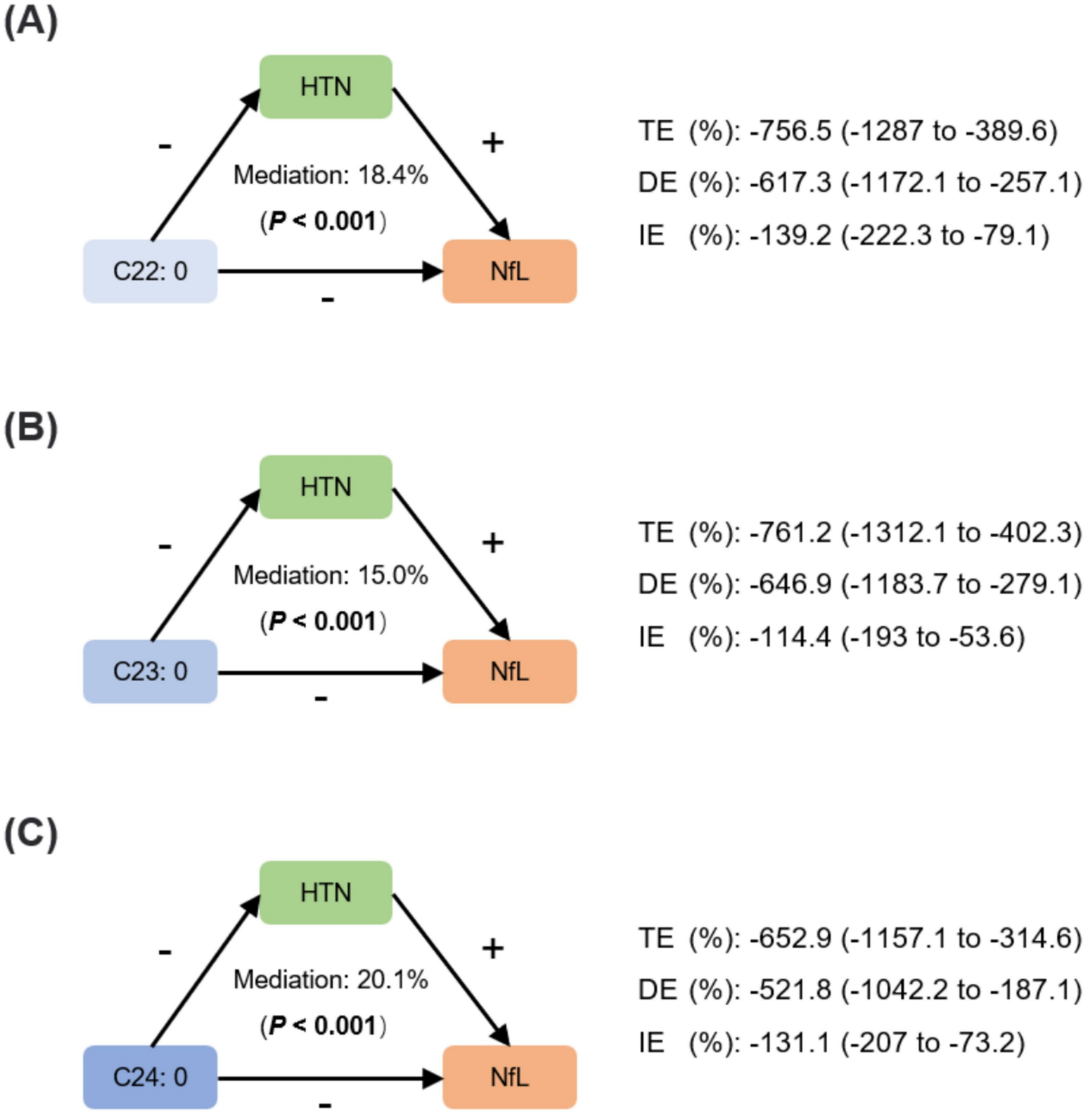
Mediating effects of hypertension between VLSFAs and NfL. The mediation analysis assessed the role of hypertension between VLSFAs and NfL, including C22:0 **(A)**, C23:0 **(B)**, and C24:0 **(C)**. The mediation proportion and statistical significance are shown, accompanied by coefficients of TE, DE, and IE. “+” or “−” indicates the positive or negative associations between VLSFAs, hypertension, and NfL. The models were adjusted for covariates, including age, sex, race/ethnicity, BMI, educational attainment, poverty income ratio, marital status, smoking status, alcohol consumption, leisure-time physical activity, Healthy Eating Index-2015, fasting blood glucose, triglyceride, and serum creatinine. TE indicates total effect; DE, direct effect; IE, indirect effect; HTN, hypertension; VLSFA, very-long-chain saturated fatty acid; NfL, neurofilament light chain.

The mediation analysis was conducted under a causal assumption that VLSFAs attenuate neurodegeneration through an underlying antihypertensive mechanism. Figure 6 showed the pathways between VLSFAs, NfL, and hypertension with labels indicating their positive or negative associations. The indirect effects through hypertension accounted for approximately 18.4% of the association between C22:0 and NfL, 15.0% between C23:0 and NfL, and 20.1% between C24:0 and NfL (all *p* values for mediation < 0.001).

### 3.9 Sensitivity analyses

The robustness of current findings was confirmed by several sensitivity analyses. First, when additional participants with missing NfL data were included (*N* = 3,192), the association between VLSFAs and hypertension persisted (Supplementary Table S7). Second, positive correlations were found between dietary FA intakes (C10:0, C12:0, C14:0, C18:1n-7, C18:3n-3, C20:4n-6, C20:5n-3, and C22:6n-3) and their corresponding circulating concentrations, suggesting the potential efficacy of dietary FA interventions to prevent neurodegeneration (Supplementary Table S8). Third, the association between VLSFAs and NfL remained after the adjustment for dietary FA intake (saturated, monounsaturated, and polyunsaturated) (Supplementary Table S9).

## 4 Discussion

In this cross-sectional study, we investigated the association between circulating fatty acids (FA) profiles and serum neurofilament light chain (NfL), a biomarker of neurodegeneration. Our findings revealed that lower levels of circulating VLSFAs were associated with higher serum NfL concentrations, with C22:0 and C23:0 contributing substantial negative weights. In this association, hypertension mediated approximately 15–20% of the indirect effects. Subgroup analyses and correlation analyses suggested that diet quality played an important role in this association and may be a potential intervention avenue against neurodegeneration.

The observed neuroprotective associations of very-long-chain saturated fatty acids (VLSFAs) likely arise from a combination of mechanisms. Our finding that hypertension partially mediates the VLSFA-NfL relationship (accounting for approximately 15–20% of the indirect effect) supports an indirect neuroprotective pathway via improved cardiovascular health. Contextualizing this magnitude is informative; while direct comparisons across studies are complex due to methodological heterogeneity, the substantial impact of hypertension on neurodegenerative risk is well-established. For instance, a large UK cohort study (Zheng et al., 2022) quantified this risk, reporting a 10% increase in incident dementia associated with hypertension in patients with type 2 diabetes (HR 1.10, 95% CI 1.03–1.18). Although representing a direct risk estimate rather than a mediation proportion, this finding underscores the clinical relevance of blood pressure control in neurodegeneration. Our estimate that 15–20% of the VLSFA-NfL association operates via hypertension aligns conceptually with this perspective, indicating that the vascular pathway is a meaningful, albeit partial, contributor to the overall observed VLSFA effect. By potentially contributing to lower blood pressure, VLSFAs may mitigate cerebrovascular damage and associated neuronal injury.

Beyond these vascular benefits, direct neurobiological roles for VLSFAs are also strongly indicated. Specific VLSFAs, particularly odd-chain species like C23:0, are crucial structural components of neuronal membranes and myelin, notably within glycosphingolipids (Svennerholm and Stallberg-Stenhagen, 1968). Adequate levels of these fatty acids are essential for maintaining myelin integrity, neuronal membrane stability, and the function of specialized membrane domains. The pronounced protective effect of C23:0 observed at lower concentrations in our study may reflect the fulfillment of such fundamental structural or functional requirements within the nervous system, consistent with its significant presence in brain lipids. This aligns with longitudinal findings from the Atherosclerosis Risk in Communities (ARIC) cohort, where higher midlife VLSFAs were linked to attenuated cognitive decline decades later (Li et al., 2020), suggesting long-term direct effects on brain resilience.

Collectively, these lines of evidence suggest that VLSFAs may confer neuroprotection through both indirect (cardiovascular-mediated) and direct (neural-structural/functional) pathways. The interplay and relative importance of these mechanisms likely vary and warrant further investigation to fully elucidate the neuroprotective profile of VLSFAs.

The health-promoting benefits of polyunsaturated fatty acids, particularly omega-3 and omega-6 FAs, have been widely recognized in the relation to cardiovascular diseases, cancer, and autoimmune diseases (Kapoor et al., 2021). Instead, it is recommended to reduce the intake of saturated FAs, as they are regarded to be detrimental to health (Helgadottir et al., 2022). However, this does not seem to be the case with circulating VLSFAs (more than 20 carbons, without double bonds) (Lemaitre and King, 2022). Extensive cardiovascular benefits of VLSFAs have been demonstrated in heart failure (Lemaitre et al., 2018; Flock and Kris-Etherton, 2013), atrial fibrillation (Fretts et al., 2014), coronary artery disease (Malik et al., 2015; Papandreou et al., 2019), and sudden cardiac arrest (Lemaitre et al., 2014). Particularly, Li et al. found that circulating VLSFAs was negatively associated with the risk of pregnancy-induced hypertension and BP levels (Li et al., 2020). Consistently, our study reveals a negative joint association between VLSFAs and hypertension in the FA profiles. This association remains between the independent FAs (C22:0, C23:0, and C24:0) and hypertension. These findings verify a potential antihypertensive effect of VLSFAs.

In addition to cardiovascular benefits, VLSFAs also exhibit potential neuroprotective effects. In the ARIC study, Li et al. were the first to report that midlife circulating VLSFAs were negatively associated with cognitive decline 20 years later, as assessed by three cognitive tests: the Delayed Word Recall Test (DWRT), the Digit-Symbol Substitution Test (DSST), and the Word Fluency Test (WFT). While foundational, this longitudinal work primarily focused on functional outcomes and did not incorporate molecular biomarkers of neurodegeneration or explore specific mediating pathways. Our study significantly extends these findings in several key ways. First, using a cross-sectional design in the NHANES cohort, we corroborate the association between higher VLSFA levels and better cognitive performance, particularly in processing speed (DSST) and memory (delayed recall) domains (Supplementary Table S4). Second, and critically, we provide direct molecular evidence by demonstrating an inverse association between VLSFAs and serum NfL (Table 2), a validated biomarker of neuroaxonal damage. This integration of both molecular (NfL) and functional (cognitive performance) assessments provides a more comprehensive picture of VLSFAs’ potential neuroprotective role than previously available. Third, as a key mechanistic advancement, our mediation analysis suggests that the protective association of VLSFAs may be partially channeled through pathways involving lower hypertension risk (Figure 6), offering a potential biological explanation for the observed benefits. These results, demonstrating associations with both NfL concentrations and cognitive performance, underscore the potential importance of VLSFAs in maintaining neurological integrity.

Given that cognitive decline is often accompanied by structural neurodegeneration (Sokolov et al., 2020), our dual-outcome approach offers a more comprehensive understanding of this relationship (Table 2; Supplementary Tables S4, S5).

Hypertension has been recognized as a major cause of age-related cognitive impairment and accelerates the process of neurodegeneration (Li et al., 2020). In the Reasons for Geographic and Racial Differences in Stroke (REGARDS) study (Levine et al., 2019), Levine et al. found that elevated systolic blood pressure (SBP), but not diastolic blood pressure (DBP), was associated with an accelerated cognitive decline over an 8-year period, suggesting a more detrimental effect of elevated SBP on neurodegeneration. Consistently, our study confirmed a positive association between hypertension and NfL, which was more pronounced with increasing SBP levels (Table 2).

Several potential genetic and behavioral factors may account for the association between VLSFAs and neurodegeneration. First, the circulating levels of VLSFAs have been found to be partially determined by genetics (Lemaitre et al., 2015), which further influences the development of diseases such as type 2 diabetes (Luo et al., 2021). Therefore, the association between VLSFAs and neurodegeneration may also be influenced by genetic background. Second, unhealthy dietary patterns have been identified as critical factors contributing to neurodegeneration (Wieckowska-Gacek et al., 2021) and cognitive aging (McGrattan et al., 2019). Additionally, circulating levels of VLSFA are closely related to dietary patterns (Pfeuffer and Jaudszus, 2016). Therefore, low levels of VLSFA are indicative of an unhealthy diet, which may exacerbate the risk of neurodegeneration.

The consistent findings between NfL (Table 2) and cognitive function assessments (Supplementary Tables S4, S5) in our study have significant clinical implications. While NfL serves as a sensitive molecular marker for neurodegeneration, as demonstrated by its association with cognitive decline (Supplementary Table S5), cognitive function tests directly reflect the functional impact of neurological changes. The observation that very-long-chain saturated fatty acids (VLSFAs) show beneficial associations with both outcomes suggests that these fatty acids may influence neurological health through multiple pathways. This comprehensive evidence highlights the potential value of VLSFA-focused interventions in preserving both the structural and functional aspects of neurological health.

There are notable findings in our study that warrant attention. First, dietary behaviors may play a crucial role in association between VLSFAs and NfL. On the one hand, we found that the majority of circulating FA concentrations exhibit a significant positive correlation with the dietary intake of corresponding FAs (Supplementary Table S8). On the other hand, compared to individuals with good diet quality, individuals with poor diet quality obtained more pronounced benefits from elevated concentrates of C23:0 (P for interaction = 0.001) and C24:0 (P for interaction = 0.035) (Figure 4). In support of this, Lai et al. found that dietary pattern with higher saturated fat intake and nut consumption contributed to a higher concentration of circulating C22:0 and C24:0 (Lai et al., 2023). Besides, higher dietary intake of VLSFAs is also associated lower risk of diseases such as metabolic syndrome (Lee et al., 2015). Therefore, improving dietary patterns is expected to be an avenue to prevent neurodegeneration. Regarding potential clinical implications, our findings suggest avenues for targeted strategies, although definitive recommendations require further investigation, particularly through intervention trials. The observed non-linear association between VLSFAs and NfL, characterized by a more pronounced protective effect at lower circulating concentrations (Figures 2, 3A), suggests a potential threshold effect. This implies that interventions aimed at increasing VLSFA levels might be most beneficial for individuals with initially low circulating concentrations. While our study did not determine optimal intake levels, the data suggest that achieving levels above the lower range (e.g., moving individuals from the lowest quartile or below the median toward higher concentrations) could be associated with reduced markers of neuroaxonal damage. Furthermore, the subgroup analysis indicating potentially greater benefits from higher C23:0 and C24:0 levels among individuals with poorer diet quality (low Healthy Eating Index-2015 score, Figure 4) tentatively identifies this group as a potential target population for future dietary interventions focusing on VLSFA-rich food sources. However, translating these circulating levels into specific dietary recommendations necessitates further research to understand the dose–response relationship between dietary VLSFA intake and resulting circulating concentrations, as well as their ultimate impact on neurological outcomes in prospective and interventional settings.

Second, compared with C22:0 and C24:0, we found a more pronounced and notably non-linear association between C23:0 and NfL. Specifically, when circulating C23:0 levels were relatively low (e.g., below the median), NfL concentrations exhibited a significant increase with further decreases in C23:0. This observation suggests a potential threshold effect, implying that C23:0 may be an essential component for maintaining the integrity of neural structures, with its protective influence being particularly critical at lower physiological concentrations. Several plausible biological mechanisms could underlie this non-linear relationship. One possibility relates to the efficiency of C23:0 incorporation into vital neural lipids or its role in specific cellular processes that become saturated or achieve optimal function at a certain threshold. For instance, as an odd-chain fatty acid, C23:0 is incorporated into complex lipids such as sphingolipids and glycerophospholipids, which are integral to neuronal membranes and myelin sheaths. At very low C23:0 availability, the synthesis or maintenance of these crucial structures might be compromised, leading to increased neuronal vulnerability and higher NfL levels. Initial supplementation or an increase in C23:0 up to a certain physiological requirement could therefore lead to a substantial restoration of membrane integrity or function, resulting in a marked decrease in NfL. However, once these structural or functional demands are met, further increases in C23:0 might yield diminishing returns in terms of NfL reduction via these specific pathways, or its effects might plateau. Svennerholm et al. highlighted the physiological relevance of odd-chain FAs within the neurological system, finding that C23:0 and C25:0 constitute a significant portion (10% of total non-hydroxylated FAs and 25% of total hydroxylated FAs) of adult human brain glycosphingolipids (Svennerholm and Stallberg-Stenhagen, 1968). This underscores the quantitative importance of C23:0 in brain lipid architecture. Given that odd-chain FAs like C23:0 are primarily obtained through dietary intake or gut microbial metabolism, our findings suggest that dietary interventions aimed at ensuring adequate C23:0 levels, particularly for individuals with lower baseline concentrations, could hold promising prospects for preventing or mitigating neurodegeneration. The pronounced effect observed at lower C23:0 levels may indeed point toward a fundamental requirement that, once fulfilled, provides a significant degree of neuroprotection.

This study has several limitations. First, the cross-sectional design precludes establishing temporal sequences between VLSFAs, hypertension, NfL, and cognition. Consequently, causal inferences cannot be drawn, and the possibility of reverse causality cannot be excluded. Specifically, the observed inverse association between VLSFA levels and NfL could potentially arise if the neurodegenerative process itself leads to alterations in systemic lipid metabolism that result in lower circulating VLSFA concentrations. If such a pathway exists, our findings might overestimate the protective effect of VLSFAs, or the association could, in part, reflect the metabolic consequences of neurodegeneration rather than a purely protective role of VLSFAs against it. Similarly, while less likely given that hypertension is often considered a precursor, it’s conceivable that advanced neurodegenerative states could influence autonomic regulation affecting both blood pressure and lipid profiles concurrently, further complicating the interpretation of the VLSFA-hypertension-NfL nexus. This lack of temporality significantly impacts the interpretation of the mediation analysis. While exploratory, the mediation findings rely on strong, untestable assumptions: specifically, the assumed temporal order (VLSFAs → hypertension → NfL/cognition) and, critically, the absence of unmeasured confounding factors that could influence all three components (e.g., specific genetic predispositions affecting both lipid metabolism and neurological health, detailed dietary patterns beyond the Healthy Eating Index-2015 (HEI-2015) score, or low-grade systemic inflammation). Therefore, the mediation results (15–20% effect) should be viewed cautiously as hypothesis-generating regarding potential pathways, rather than conclusive evidence. Second, while we focused on age-related neurodegeneration markers, NHANES data does not allow for the complete exclusion of specific underlying neurological conditions that might affect NfL levels. Third, the use of an unweighted sample, chosen to facilitate the complex mixture modeling, may limit the direct generalizability of the quantitative estimates to the entire US population, although the NHANES sampling design itself aims for national representation. Fourth, while NfL is a valuable marker of neuroaxonal damage, it lacks pathological specificity and cannot localize damage without complementary data like neuroimaging.

## 5 Conclusion

Our serum lipidomic analysis identified very-long-chain saturated fatty acids (VLSFAs: C22:0, C23:0, and C24:0) as potential therapeutic targets for neurodegeneration, demonstrating significant inverse associations with serum neurofilament light chain (NfL) levels and positive correlations with cognitive performance. The stronger protective association at lower VLSFA concentrations suggests a clinically relevant threshold effect. Mechanistic investigation revealed hypertension as a partial mediator, accounting for approximately 15–20% of the relationship between VLSFAs and neurodegeneration biomarkers. These findings highlight dual neuroprotective pathways through which these lipid species may confer benefits. Further interventional studies are warranted to determine whether targeted strategies (e.g., dietary modifications) aimed at modulating circulating VLSFA levels, particularly in individuals with lower baseline concentrations or poorer diet quality, can effectively mitigate neurodegeneration and enhance cognitive function.

## Data Availability

The original contributions presented in the study are included in the article/Supplementary material, further inquiries can be directed to the corresponding author.

## Glossary

NfL: Neurofilament light chain
VLSFA: Very-long-chain saturated fatty acid
FA: Fatty acid
ARIC: Atherosclerosis risk in communities
NHANES: National Health and Nutrition Examination Survey
eGFR: Estimated glomerular filtration rate
LLOD: Lower limit of detection
BP: Blood pressure
SBP: Systolic blood pressure
DBP: Diastolic blood pressure
PA: Physical activity
PIR: Poverty income ratio
BMI: Body mass index
HEI-2015: Healthy eating index-2015
Lowess: Locally weighted scatterplot smoothing
RCS: Restricted cubic spline
FBG: Fasting blood glucose
WQS: Weighted quantile sum
BKMR: Bayesian kernel machine regression
Qgcomp: Quantile g-computation
IE: Indirect effect
DE: Direct effect
TE: Total effect
DWRT: Delayed word recall test
DSST: Digit-symbol substitution test
WFT: Word fluency test
REGARDS: Reasons for geographic and racial differences in stroke
