# Supplementary material for: Serum lipidomic analysis identifies potential therapeutic targets for neurodegeneration
Source: Front Hum Neurosci. 2025 Jun 18;19:1598495. doi: 10.3389/fnhum.2025.1598495 (PMC12213555; doi:10.3389/fnhum.2025.1598495)
Supplement: Supplementary file 1 [file Data_Sheet_1.docx]

# Supplementary Materials

**Serum Lipidomic Analysis Identifies Potential Therapeutic Targets for Neurodegeneration**

*Lumi Zhang^1†^, Duanbin Li^2†^, Na Zhao^1*^, Guoping Peng^3*^*

*^1^ Department of Neurology, Wenzhou TCM Hospital of Zhejiang Chinese Medical University, Wenzhou, China.*

*^2^ Department of Cardiology, Sir Run Run Shaw Hospital, School of Medicine, Zhejiang University, Hangzhou, China.*

*^3^ Department of Neurology, The First Affiliated Hospital, Zhejiang University School of Medicine, Hangzhou, China.*

^†^ Lumi Zhang and Duanbin Li contributed equally to this work.

**Short title**: VLSFAs, hypertension, and Neurodegeneration

*Correspondence to:

Prof. Guoping Peng

E-mail: guopingpeng@zju.edu.cn

Prof. Na Zhao

E-mail: [zhaonawz82@163.com](mailto:zhaonawz82@163.com)

**Figure S1.** Flowchat

**Figure S2.** Concentration distribution of circulating FA profiles

**Figure S3.** Correlation matrix of circulating FA profiles

**Figure S4.** Association between circulating FA profiles and hypertension

**Figure S5.** Assessment of the joint association between VLSFAs and hypertension using BKMR and Qgcomp models

**Figure S6.** Subgroup analyses between VLSFAs and hypertension

**Table S1.** Lower limit of detection and detection rate of FA profiles

**Table S2.** Healthy Eating Index-2015 components and scoring standards

**Table S3.** Baseline characteristics of FA profiles according to serum NfL levels

**Table S4.** Association between VLSFAs and cognitive function in a subset population (N = 418, age ≥ 60 years)

**Table S5.** Association between NfL and cognitive function in a subset population (N = 418, age ≥ 60 years)

**Table S6.** Population quartiles by the concentration of VLSFAs (N = 1677)

**Table S7.** Association between VLSFAs and hypertension in an extended population (N = 3192)

**Table S8.** Correlation between circulating FA concentrations and dietary FA intakes in a subset population (N = 1398)

**Table S9.** Association between VLSFAs and NfL concentration with further adjustment for dietary FA intakes (N = 1398)

**Figure S1.** Flowchat


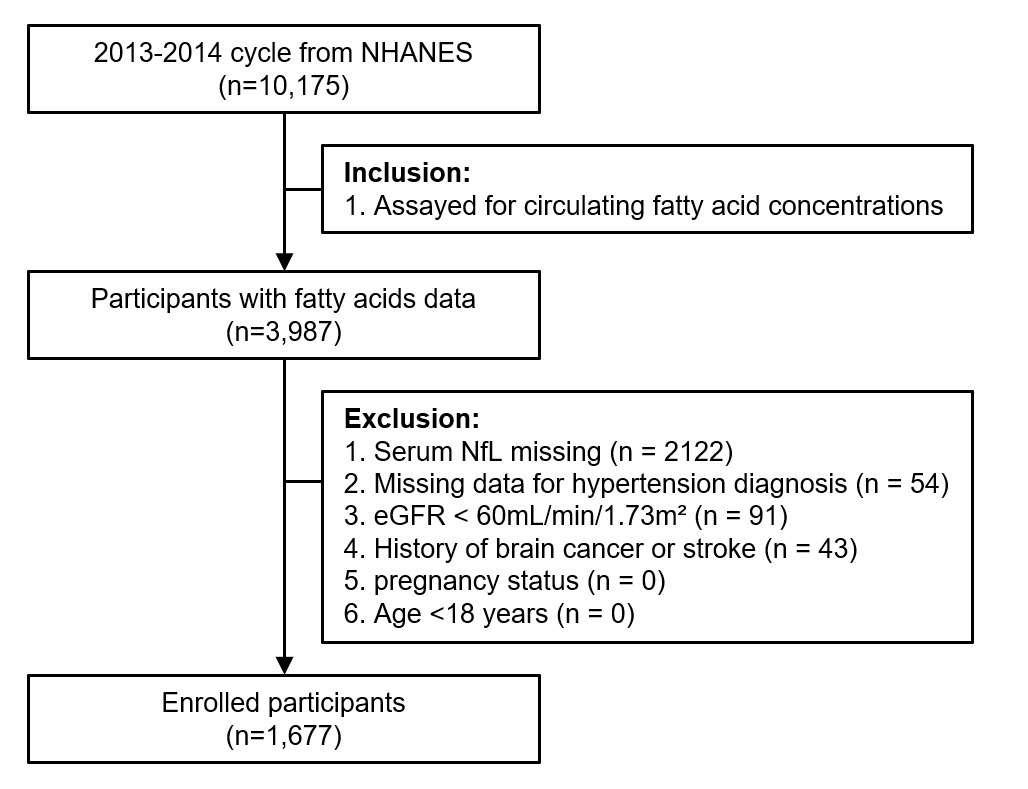


**Figure S2.** Concentration distribution of circulating FA profiles


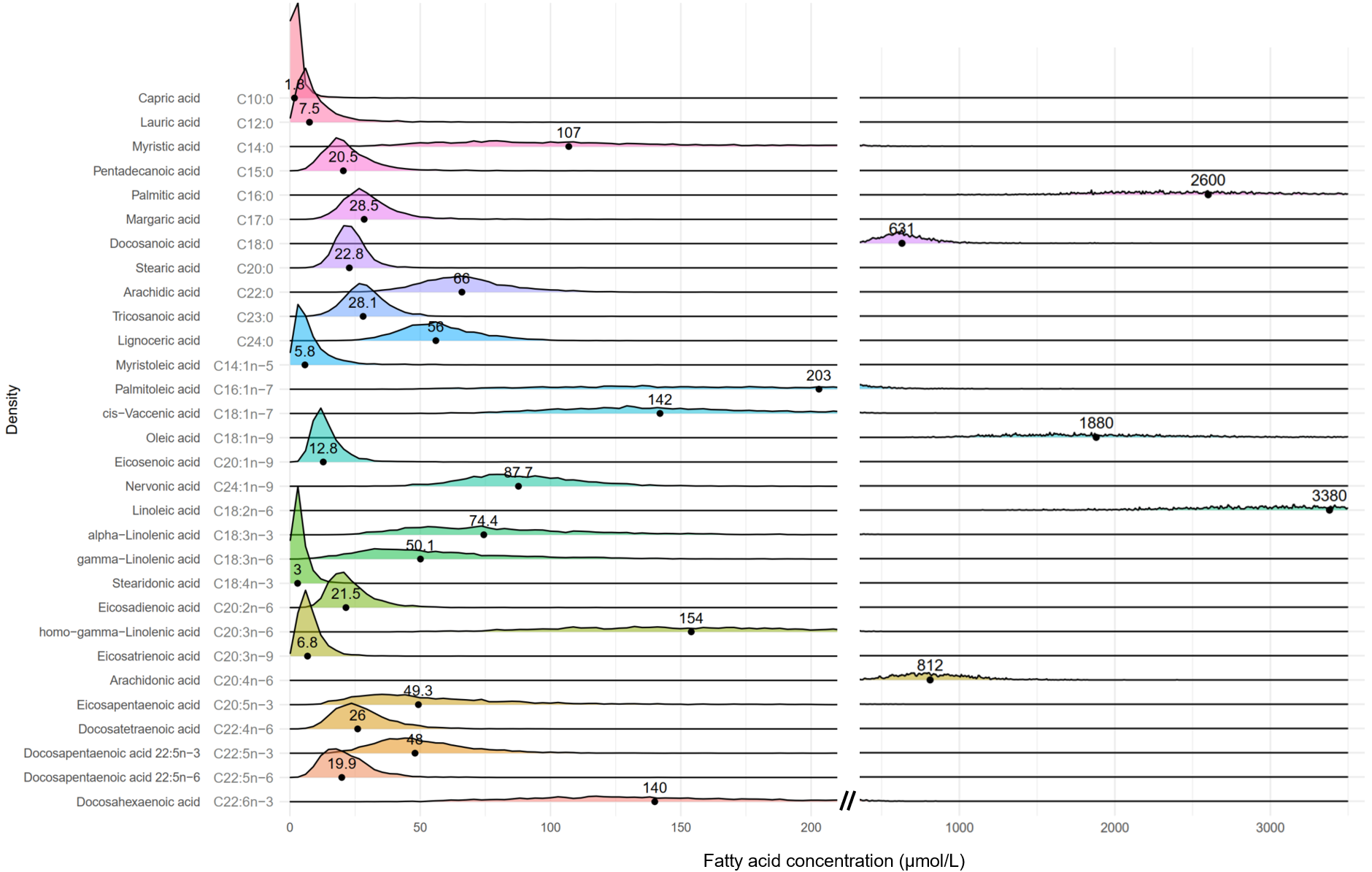


The ridge plot shows the concentration distribution of 30 different fatty acids. Each fatty acid is marked with a point indicating its median concentration, accompanied by the exact numerical value.

**Figure S3.** Correlation matrix of circulating FA profiles


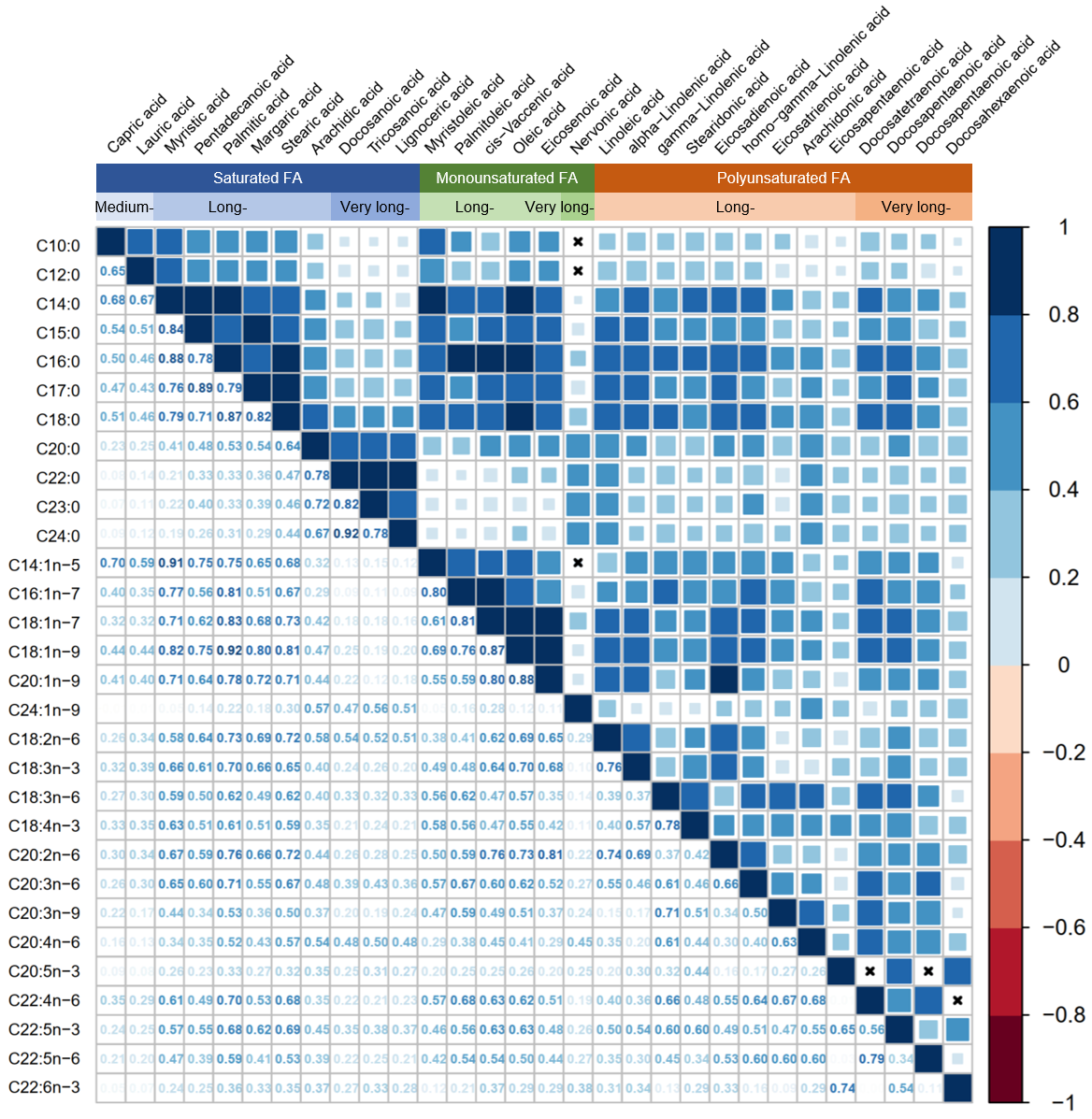


The concentration of fatty acids is subjected to a natural logarithm transformation before correlation analysis. Pearson correlation coefficients are shown in the lower left corner. The squares in the upper right corner illustrate the magnitude of correlation, with larger squares indicating stronger correlations. Blue indicates positive correlations, while red indicates negative correlations. All correlations between fatty acids show statistical significance (P value < 0.05), except for those marked with a cross.

**Figure S4.** Association between circulating FA profiles and hypertension


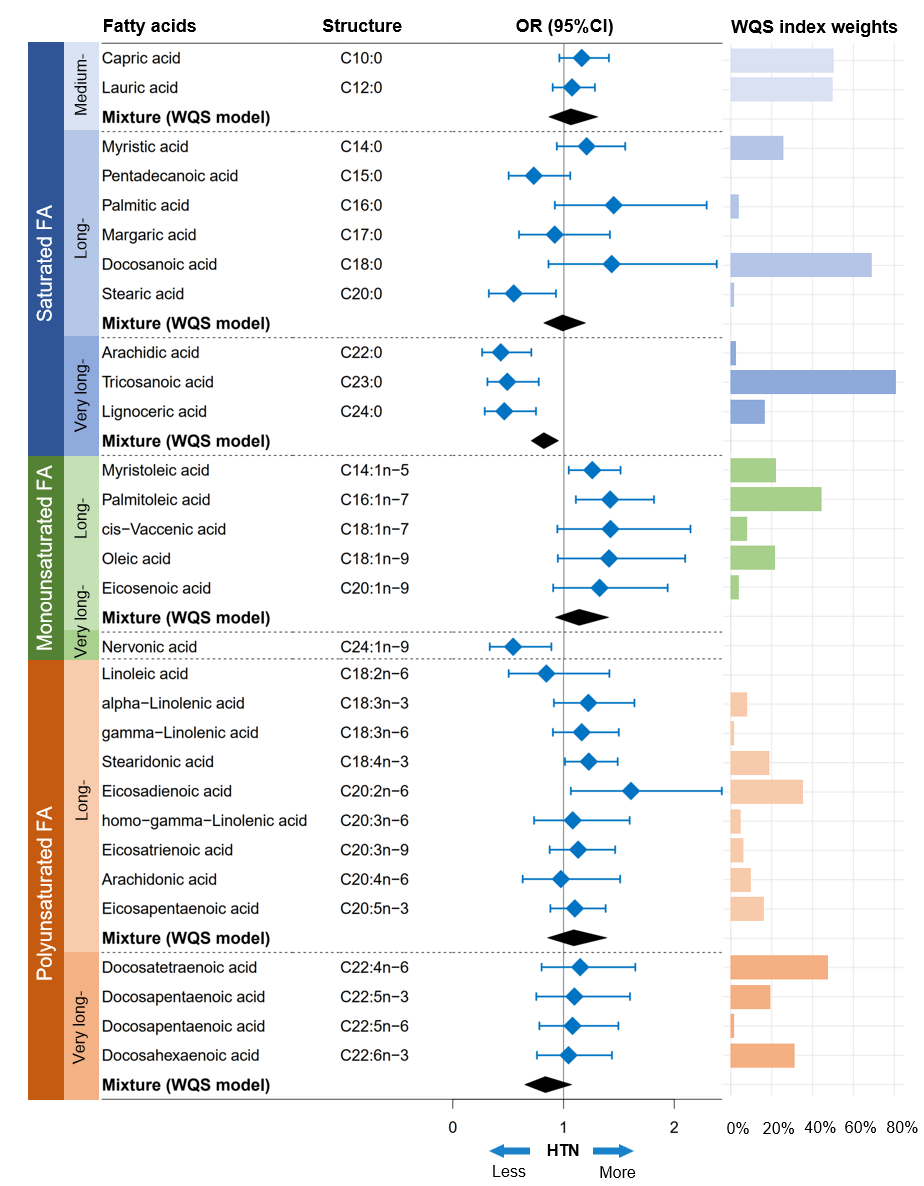


Independent associations between FAs and hypertension were estimated using logistic regression models. Joint associations between FA subtypes and hypertension were estimated using WQS models. All models were adjusted for covariates, including age, sex, race/ethnicity, BMI, educational attainment, PIR, marital status, smoking status, alcohol consumption, leisure-time PA, HEI-2015 score, FBG, triglyceride, serum creatinine.

**Figure S5.** Assessment of the joint association between VLSFAs and hypertension using BKMR and Qgcomp models


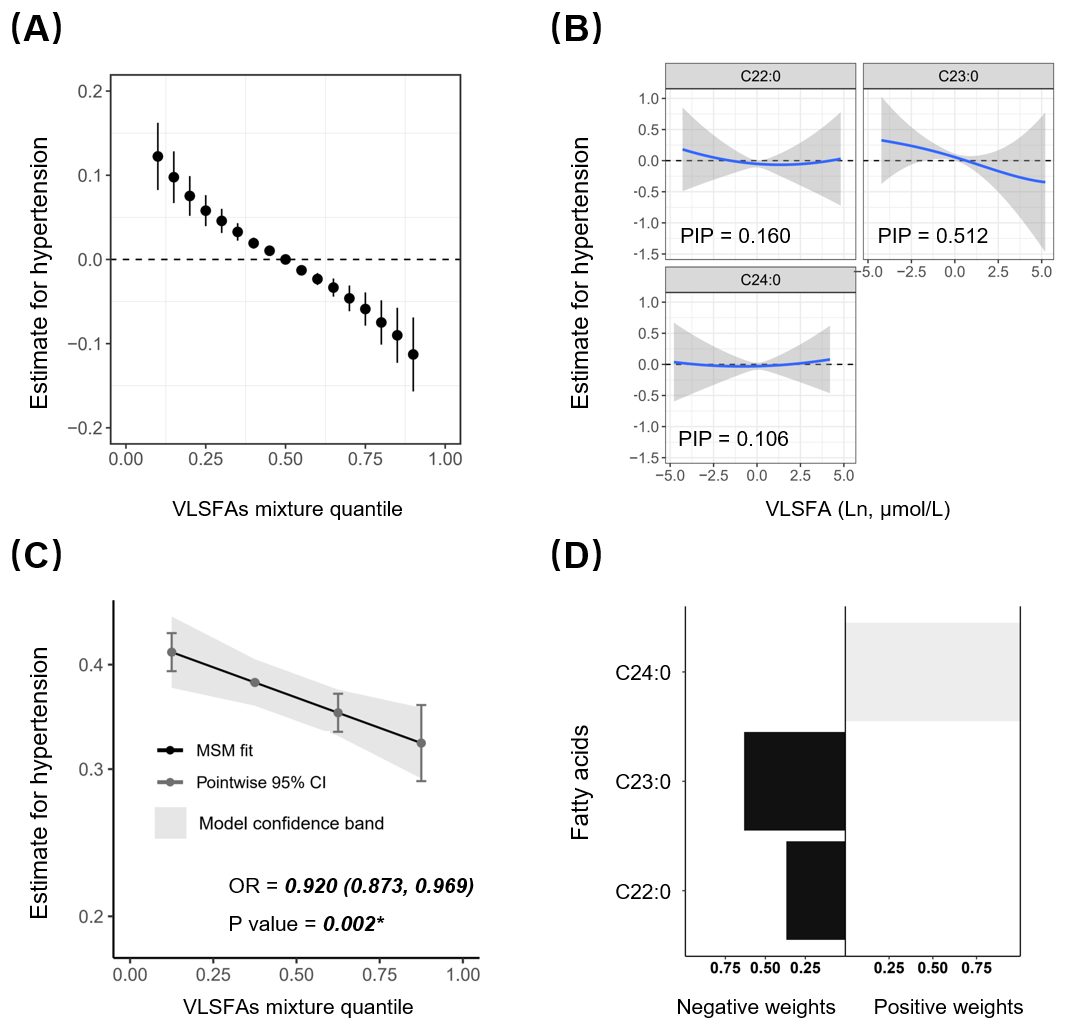


The BKMR model presents estimates with 95% CIs for joint VLSFAs associated with hypertension (A). All the VLSFAs at certain percentiles (increment by 0.05) are compared to the VLSFAs at the 50th percentile. In addition, dose-response associations of independent VLSFA with hypertension are also presented when fixing remaining VLSFAs concentrations (B). In BKMR model, posterior inclusion probability (PIP) serves as an indicator of the relative importance of each component in joint VLSFAs, with greater values indicating higher importance. Qgcomp model further quantify and visualize the association between joint VLSFAs and hypertension (C), as well as the positive or negative weights of each VLSFAs component (D). All models were adjusted for covariates, including age, sex, race/ethnicity, BMI, educational attainment, PIR, marital status, smoking status, alcohol consumption, leisure-time PA, HEI-2015 score, FBG, triglyceride, serum creatinine.

**Figure S6.** Subgroup analyses between VLSFAs and hypertension


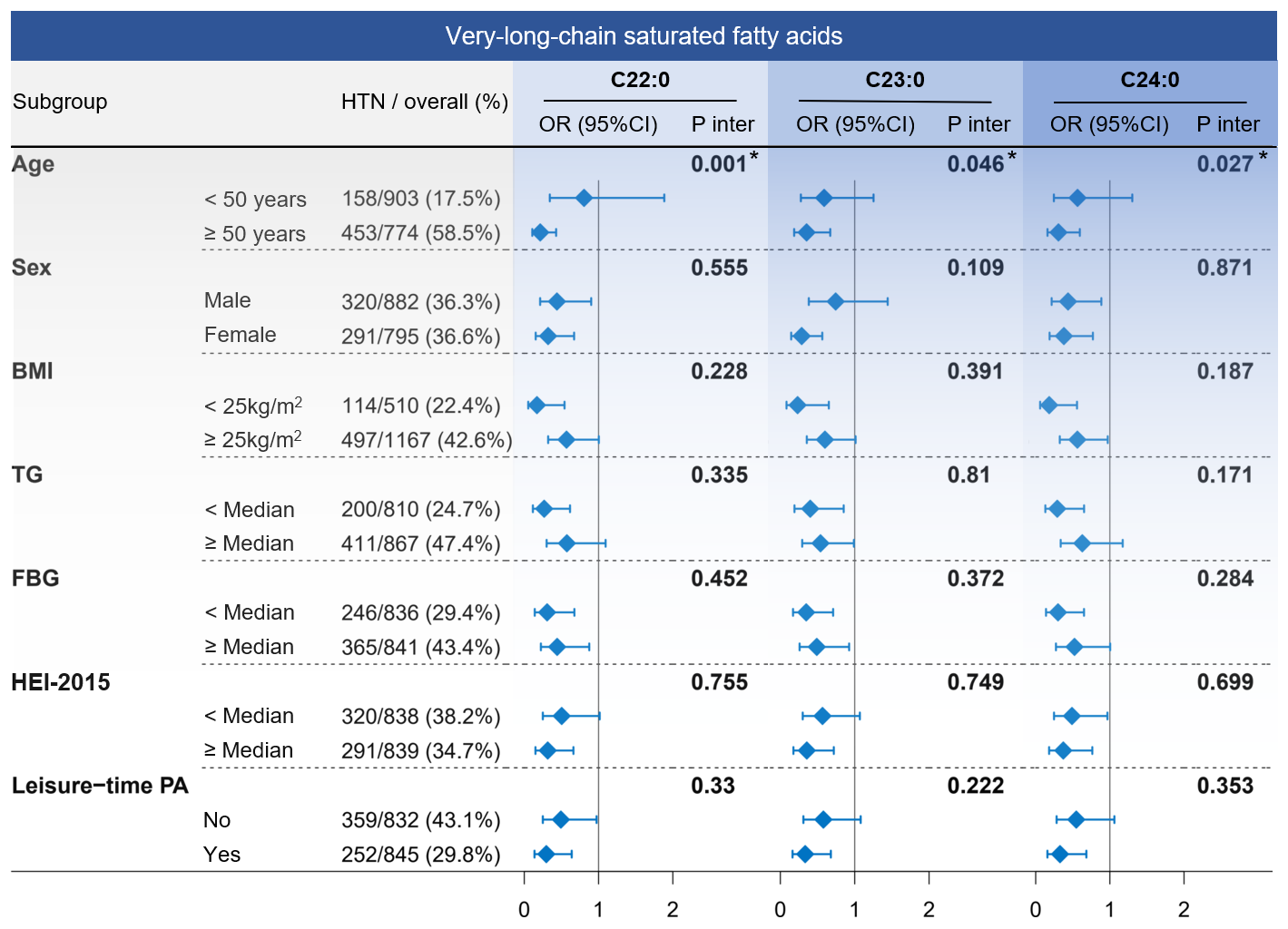


The association between circulating VLSFAs and hypertension was estimated when stratified by age, sex, BMI, TG, FBG, HEI-2015, and leisure-time PA. The median values of TG, FBG, and HEI-2015 were 1.06 mmol/L, 5.27 mmol/L, and 50.8, respectively. Models were adjusted for covariates, including age, sex, race/ethnicity, BMI, educational attainment, PIR, marital status, smoking status, alcohol consumption, leisure-time PA, HEI-2015, FBG, triglyceride, serum creatinine (except for the stratified variable itself).

**Table S1.** Lower limit of detection and detection rate of FA profiles

| Fatty Acids | Structure | LLOD  (μmol/L) | At or above the detection limit | Below lower detection limit | Detection rate (%) |
| --- | --- | --- | --- | --- | --- |
| Capric acid | C10:0 | 1.59 | 931 | 746 | 55.5 |
| Lauric acid | C12:0 | 2.33 | 1631 | 46 | 97.3 |
| Myristic acid | C14:0 | 4.9 | 1677 | 0 | 100 |
| Pentadecanoic acid | C15:0 | 0.75 | 1677 | 0 | 100 |
| Palmitic acid | C16:0 | 78.1 | 1677 | 0 | 100 |
| Margaric acid | C17:0 | 3.36 | 1677 | 0 | 100 |
| Stearic acid | C18:0 | 39.1 | 1677 | 0 | 100 |
| Arachidic acid | C20:0 | 0.82 | 1677 | 0 | 100 |
| Docosanoic acid | C22:0 | 0.68 | 1677 | 0 | 100 |
| Tricosanoic acid | C23:0 | 0.9 | 1677 | 0 | 100 |
| Lignoceric acid | C24:0 | 1.09 | 1677 | 0 | 100 |
| Myristoleic acid | C14:1n-5 | 0.29 | 1677 | 0 | 100 |
| Palmitoleic acid | C16:1n-7 | 6.56 | 1677 | 0 | 100 |
| cis-Vaccenic acid | C18:1n-7 | 2.31 | 1677 | 0 | 100 |
| Oleic acid | C18:1n-9 | 17.7 | 1677 | 0 | 100 |
| Eicosenoic acid | C20:1n-9 | 0.87 | 1677 | 0 | 100 |
| Nervonic acid | C24:1n-9 | 0.69 | 1677 | 0 | 100 |
| Linoleic acid | C18:2n-6 | 22.6 | 1677 | 0 | 100 |
| alpha-Linolenic acid | C18:3n-3 | 1.54 | 1677 | 0 | 100 |
| gamma-Linolenic acid | C18:3n-6 | 0.42 | 1677 | 0 | 100 |
| Stearidonic acid | C18:4n-3 | 0.24 | 1675 | 2 | 99.9 |
| Eicosadienoic acid | C20:2n-6 | 0.31 | 1677 | 0 | 100 |
| homo-gamma-Linolenic acid | C20:3n-6 | 1.14 | 1677 | 0 | 100 |
| Eicosatrienoic acid | C20:3n-9 | 0.39 | 1677 | 0 | 100 |
| Arachidonic acid | C20:4n-6 | 7.34 | 1677 | 0 | 100 |
| Eicosapentaenoic acid | C20:5n-3 | 0.79 | 1677 | 0 | 100 |
| Docosatetraenoic acid | C22:4n-6 | 0.31 | 1677 | 0 | 100 |
| Docosapentaenoic acid | C22:5n-3 | 0.55 | 1677 | 0 | 100 |
| Docosapentaenoic acid | C22:5n-6 | 0.24 | 1677 | 0 | 100 |
| Docosahexaenoic acid | C22:6n-3 | 1.84 | 1677 | 0 | 100 |

**Table S2.** Healthy Eating Index-2015 components and scoring standards

| Component | Maximum points | Standard for maximum score | Standard for minimum score of zero |
| --- | --- | --- | --- |
| **Adequacy** |  |  |  |
| Total Fruits | 5 | 0.8 c equivalents/1,000 kcal | No fruit |
| Whole Fruits | 5 | 0.4 c equivalents/1,000 kcal | No whole fruit |
| Total Vegetables | 5 | 1.1 c equivalents/1,000 kcal | No vegetables |
| Greens and Beans | 5 | 0.2 c equivalents/1,000 kcal | No dark green vegetables or beans and peas |
| Whole Grains | 10 | 1.5 oz equivalents/1,000 kcal | No whole grains |
| Dairy | 10 | 1.3 c equivalents/1,000 kcal | No dairy |
| Total Protein Foods | 5 | 2.5 oz equivalents/1,000 kcal | No protein foods |
| Seafood and Plant Proteins | 5 | 0.8 c equivalents/1,000 kcal | No seafood or plant proteins |
| Fatty Acids | 10 | (PUFAs^a^+MUFAs^b^)/SFAs^c^ >2.5 | (PUFAs^a^+MUFAs^b^)/SFAs^c^ <1.2 |
| **Moderation** |  |  |  |
| Refined Grains | 10 | <1.8 oz equivalents/1,000 kcal | 4.3 oz equivalents/1,000 kcal |
| Sodium | 10 | <1.1 g/1,000 kcal | 2.0 g/1,000 kcal |
| Added Sugars | 10 | 6.5% of energy | 26% of energy |
| Saturated Fats | 10 | 8% of energy | 16% of energy |

^a^PUFAs, polyunsaturated fatty acids; ^b^MUFAs, monounsaturated fatty acids; ^c^SFAs, saturated fatty acids.

**Table S3.** Baseline characteristics of FA profiles according to serum NfL levels

| Fatty Acids (μmol/L) | Structure | Overall | Median of serum NfL | | P value |
| --- | --- | --- | --- | --- | --- |
|  |  |  | < 12.4 pg/mL | ≥ 12.4 pg/mL |  |
| Capric acid | C10:0 | 1.76 [1.12, 2.86] | 1.64 [1.12, 2.76] | 1.84 [1.12, 2.95] | 0.018 |
| Lauric acid | C12:0 | 7.51 [4.80, 12.90] | 7.04 [4.61, 12] | 7.87 [4.96, 13.70] | 0.007 |
| Myristic acid | C14:0 | 107 [74, 159] | 100 [70.38, 149.25] | 113 [78.80, 170] | <0.001 |
| Pentadecanoic acid | C15:0 | 20.50 [16.10, 26.80] | 20.10 [15.90, 25.52] | 20.80 [16.40, 28.10] | 0.018 |
| Palmitic acid | C16:0 | 2600 [2140, 3220] | 2510 [2077.50, 3052.50] | 2690 [2220, 3340] | <0.001 |
| Margaric acid | C17:0 | 28.50 [23.50, 35] | 27.75 [23.10, 33.42] | 29.10 [24, 35.70] | 0.005 |
| Stearic acid | C18:0 | 631 [541, 746] | 616.50 [533.75, 726.25] | 644 [549, 764] | 0.001 |
| Arachidic acid | C20:0 | 22.80 [19.30, 26.40] | 22.70 [19.40, 26.10] | 22.90 [19.30, 26.90] | 0.315 |
| Docosanoic acid | C22:0 | 66 [55.90, 76.50] | 66.40 [57.10, 75.73] | 64.90 [54.40, 77.90] | 0.235 |
| Tricosanoic acid | C23:0 | 28.10 [23.80, 33.40] | 28.50 [24.10, 32.80] | 28 [23.30, 34.10] | 0.536 |
| Lignoceric acid | C24:0 | 56 [47.20, 66] | 56.15 [47.98, 65.12] | 55.60 [46.50, 67.10] | 0.715 |
| Myristoleic acid | C14:1n-5 | 5.77 [3.53, 10.10] | 5.36 [3.33, 9.14] | 6.36 [3.68, 11] | <0.001 |
| Palmitoleic acid | C16:1n-7 | 203 [134, 306] | 183 [125, 276.25] | 218 [144, 334] | <0.001 |
| cis-Vaccenic acid | C18:1n-7 | 142 [117, 180] | 138 [112, 168] | 149 [123, 189] | <0.001 |
| Oleic acid | C18:1n-9 | 1880 [1500, 2420] | 1790 [1420, 2270] | 1990 [1590, 2600] | <0.001 |
| Eicosenoic acid | C20:1n-9 | 12.80 [10.30, 16.60] | 12.20 [9.75, 15.80] | 13.40 [10.80, 17.50] | <0.001 |
| Nervonic acid | C24:1n-9 | 87.70 [74.60, 103] | 87.30 [75.35, 102] | 88.20 [73.90, 106] | 0.361 |
| Linoleic acid | C18:2n-6 | 3380 [2850, 3960] | 3400 [2927.50, 3952.50] | 3380 [2740, 3970] | 0.092 |
| alpha-Linolenic acid | C18:3n-3 | 74.40 [54.50, 103] | 72.95 [53.50, 101] | 76.30 [55.20, 108] | 0.096 |
| gamma-Linolenic acid | C18:3n-6 | 50.10 [34.60, 72.20] | 46.75 [32.88, 66.15] | 54.90 [36.80, 79.60] | <0.001 |
| Stearidonic acid | C18:4n-3 | 2.96 [1.79, 4.81] | 2.62 [1.66, 4.20] | 3.31 [1.96, 5.26] | <0.001 |
| Eicosadienoic acid | C20:2n-6 | 21.50 [17.20, 27.10] | 20.90 [16.70, 26.30] | 22 [18.10, 27.60] | 0.001 |
| homo-gamma-Linolenic acid | C20:3n-6 | 154 [118, 194] | 153 [115, 192] | 156 [121, 196] | 0.123 |
| Eicosatrienoic acid | C20:3n-9 | 6.77 [4.72, 9.63] | 6.14 [4.32, 8.53] | 7.56 [5.26, 11] | <0.001 |
| Arachidonic acid | C20:4n-6 | 812 [669, 1000] | 784.50 [657, 973.50] | 847 [684, 1030] | <0.001 |
| Eicosapentaenoic acid | C20:5n-3 | 49.30 [33.80, 74.90] | 44.25 [31.90, 67.40] | 54.90 [37, 85.70] | <0.001 |
| Docosatetraenoic acid | C22:4n-6 | 26 [20, 33.40] | 24.80 [19.40, 32.23] | 26.90 [20.80, 34.70] | <0.001 |
| Docosapentaenoic acid | C22:5n-3 | 48 [38.20, 61.40] | 45.95 [35.77, 58.18] | 51.10 [40.90, 64.70] | <0.001 |
| Docosapentaenoic acid | C22:5n-6 | 19.90 [14.80, 26.30] | 19.45 [14.50, 25.70] | 20.30 [15.40, 26.90] | 0.031 |
| Docosahexaenoic acid | C22:6n-3 | 140 [108, 188] | 134 [104, 176] | 148 [113, 206] | <0.001 |

Continuous concentrations of FA profiles are expressed as median [interquartile range] and compared using Mann-Whitney U test

**Table S4.** Association between VLSFAs and cognitive function in a subset population (N = 418, age ≥ 60 years)

| Cognitive function test | Docosanoic acid (C22:0) | | |  | Tricosanoic acid (C23:0) | | |  | Lignoceric acid (C24:0) | | |
| --- | --- | --- | --- | --- | --- | --- | --- | --- | --- | --- | --- |
|  | Beta | 95% CI | P value |  | Beta | 95% CI | P |  | Beta | 95% CI | P value |
| CERAD: mean/total score |  |  |  |  |  |  |  |  |  |  |  |
| Recall trail 1-3 | 0.454 | (-0.066, 0.974) | 0.088 |  | 0.598 | (0.092, 1.103) | 0.021* |  | 0.558 | (0.059, 1.056) | 0.029* |
| Delayed recall trail | 0.827 | (0.020, 1.635) | 0.045* |  | 0.841 | (0.055, 1.628) | 0.037* |  | 0.774 | (-0.002, 1.550) | 0.051 |
| CERAD: mean/total intrusion word |  |  |  |  |  |  |  |  |  |  |  |
| Recall trail 1-3 | -0.086 | (-0.217, 0.046) | 0.201 |  | -0.048 | (-0.176, 0.080) | 0.463 |  | -0.053 | (-0.180, 0.073) | 0.407 |
| Delayed recall trail | 0.219 | (-0.016, 0.454) | 0.069 |  | 0.226 | (-0.004, 0.455) | 0.055 |  | 0.248 | (0.023, 0.474) | 0.032* |
| Animal Fluency test |  |  |  |  |  |  |  |  |  |  |  |
| Total score | 0.654 | (-1.433, 2.740) | 0.540 |  | 1.037 | (-0.996, 3.070) | 0.318 |  | 0.577 | (-1.429, 2.582) | 0.573 |
| Digit Symbol Substitution test (DSST) |  |  |  |  |  |  |  |  |  |  |  |
| Total score | 7.952 | (2.298, 13.606) | 0.006* |  | 8.809 | (3.311, 14.306) | 0.002* |  | 7.203 | (1.763, 12.643) | 0.010* |

To reveal the potential neuroprotective role of circulating VLSFAs, we assessed the association between circulating VLSFAs and cognitive function in a subset population undergoing cognitive function evaluation (N = 418). All models were adjusted for covariates, including age, sex, race/ethnicity, BMI, educational attainment, PIR, marital status, smoking status, alcohol consumption, leisure-time PA, HEI-2015, FBG, triglyceride, serum creatinine.

^*^P value <0.0

**Table S5.** Association between NfL and cognitive function in a subset population (N = 418, age ≥ 60 years)

| Cognitive function test | Model 1 (Crude) | | |  | Model 2 (Adjusted) | | |  | Model 3 (Adjusted) | | |
| --- | --- | --- | --- | --- | --- | --- | --- | --- | --- | --- | --- |
|  | Beta | 95% CI | P value |  | Beta | 95% CI | P |  | Beta | 95% CI | P value |
| CERAD: mean/total score |  |  |  |  |  |  |  |  |  |  |  |
| Recall trail 1-3 | -0.376 | (-0.625, -0.127) | 0.003* |  | -0.301 | (-0.545, -0.056) | 0.016* |  | -0.269 | (-0.518, -0.02) | 0.035* |
| Delayed recall trail | -0.425 | (-0.813, -0.037) | 0.033* |  | -0.322 | (-0.701, 0.057) | 0.097 |  | -0.269 | (-0.654, 0.117) | 0.173 |
| CERAD: mean/total intrusion word |  |  |  |  |  |  |  |  |  |  |  |
| Recall trail 1-3 | 0.009 | (-0.052, 0.071) | 0.768 |  | 0.006 | (-0.055, 0.068) | 0.839 |  | 0.019 | (-0.044, 0.082) | 0.557 |
| Delayed recall trail | -0.012 | (-0.122, 0.097) | 0.826 |  | -0.02 | (-0.133, 0.092) | 0.725 |  | 0 | (-0.115, 0.114) | 0.994 |
| Animal Fluency test |  |  |  |  |  |  |  |  |  |  |  |
| Total score | -1.457 | (-2.465, -0.45) | 0.005* |  | -1.345 | (-2.301, -0.389) | 0.006* |  | -1.174 | (-2.148, -0.2) | 0.019* |
| Digit Symbol Substitution test (DSST) |  |  |  |  |  |  |  |  |  |  |  |
| Total score | -4.795 | (-7.87, -1.721) | 0.002* |  | -3.457 | (-5.79, -1.124) | 0.004* |  | -2.450 | (-4.762, -0.138) | 0.038* |

To verify the role of serum NfL as a biomarker for neurodegeneration, we assessed the association between serum NfL concentrations and cognitive function in a subset population undergoing cognitive function evaluation (N = 418). A series of cognitive function assessments were introduced, including: word learning and recall modules from the Consortium to Establish a Registry for Alzheimer’s disease (CERAD), the Animal Fluency test, and the Digit Symbol Substitution test (DSST). Model 1 was adjusted for none. Model 2 was adjusted for age, sex, race/ethnicity, BMI, educational attainment, PIR, marital status. Model 3 was further adjusted for smoking status, alcohol consumption, leisure-time PA, HEI-2015, FBG, triglyceride, serum creatinine.

Each cognitive assessment has a distinct purpose: 1) CERAD Word Learning Subtest (CERAD W-L): Assesses immediate and delayed learning for new verbal information, used in major studies. It involves three trials where participants read and recall 10 words. The order changes, and the maximum score is 10 per trial. In NHANES, words are presented on a computer monitor. Delayed recall happens after other exercises, approximately 8-10 minutes later. Scores for each trial, delayed recall, and intrusion count are in the data file. 2) Animal Fluency Test: Evaluates verbal fluency, distinguishing cognitive functioning levels. Participants name as many animals as possible in one minute, with points awarded for each. In NHANES, participants practice by naming three items of clothing; those unable to continue. 3) Digit Symbol Substitution Test (DSST): Assesses processing speed, sustained attention, and working memory. Participants copy symbols corresponding to 9 numbers in 133 boxes within 2 minutes. The score is the total number of correct matches. In NHANES, participants who can't match symbols with numbers during practice do not continue. Details on scoring can be found in the 1999-2000 NHANES CFQ questionnaire data file documentation (<https://wwwn.cdc.gov/Nchs/Nhanes/1999-2000/CFQ.htm>). ^*^P value <0.05.

**Table S6.** Population quartiles by the concentration of VLSFAs (N = 1677)

| Fatty Acids | Structure | Median | IQR | Q1 | |  | Q2 | |  | Q3 | |  | Q4 | |
| --- | --- | --- | --- | --- | --- | --- | --- | --- | --- | --- | --- | --- | --- | --- |
|  |  |  |  | Range | Count |  | Range | Count |  | Range | Count |  | Range | Count |
| Docosanoic acid | C22:0 | 66 | 55.9-76.5 | [21.2, 56.0) | 421 |  | [56.0, 66.1) | 420 |  | [66.1, 76.6) | 418 |  | [76.6,231.0] | 418 |
| Tricosanoic acid | C23:0 | 28.1 | 23.8-33.4 | [ 8.32, 23.9) | 423 |  | [23.90, 28.2) | 418 |  | [28.20, 33.5) | 421 |  | [33.50,123.0] | 415 |
| Lignoceric acid | C24:0 | 56 | 47.2-66 | [15.4, 47.3) | 427 |  | [47.3, 56.1) | 413 |  | [56.1, 66.1) | 419 |  | [66.1,172.0] | 418 |

IQR indicates interquartile range; Q, Quartile.

**Table S7.** Association between VLSFAs and hypertension in an extended population (N = 3192)

| VLSFAs | Structure | OR (95% CI) for categorical VLSFAs | | | | P trend | OR (95% CI) for  continuous VLSFAs | P value |
| --- | --- | --- | --- | --- | --- | --- | --- | --- |
|  |  | Q1 | Q2 | Q3 | Q4 |  |  |  |
| Docosanoic acid | C22:0 | 1 (Reference) | 0.770 (0.549, 1.080) | 0.945 (0.683, 1.307) | 0.587 (0.426, 0.808) | 0.002* | 0.491 (0.320, 0.753) | 0.001* |
| Tricosanoic acid | C23:0 | 1 (Reference) | 0.816 (0.586, 1.135) | 0.905 (0.653, 1.255) | 0.623 (0.451, 0.860) | 0.005* | 0.559 (0.374, 0.834) | 0.004* |
| Lignoceric acid | C24:0 | 1 (Reference) | 0.745 (0.534, 1.040) | 0.733 (0.529, 1.016) | 0.670 (0.489, 0.917) | 0.024* | 0.574 (0.380, 0.869) | 0.009* |

To verify the robustness of the association between circulating VLSFAs and hypertension, we expanded the target population (N=1677) to an extended population (N = 3192) by including individuals with missing NfL data. Multivariable logistic regression models were employed, considering following covariates: age, sex, race/ethnicity, BMI, educational attainment, PIR, marital status, smoking status, alcohol consumption, leisure-time PA, HEI-2015, FBG, triglyceride, serum creatinine. VLSFA indicates very-long-chain saturated fatty acids; OR, odds ratio; CI, confidence interval; others, refer to Table 1.

^*^P value <0.05

**Table S8.** Correlation between circulating FA concentrations and dietary FA intakes in a subset population (N = 1398)

| Fatty acids | | Correlation coefficient (Rho) | P value |
| --- | --- | --- | --- |
| Saturated FA | | | |
|  | C10:0 | 0.209 | <0.001* |
|  | C12:0 | 0.171 | <0.001* |
|  | C14:0 | 0.153 | <0.001* |
|  | C16:0 | -0.019 | 0.483 |
|  | C18:0 | 0.009 | 0.745 |
| Monounsaturated FA | | | |
|  | C16:1n-7 | -0.039 | 0.144 |
|  | C18:1n-7 | 0.151 | <0.001* |
|  | C20:1n-9 | -0.026 | 0.339 |
| Polyunsaturated FA | | | |
|  | C18:2n-6 | 0.041 | 0.121 |
|  | C18:3n-3 | 0.058 | 0.030* |
|  | C18:4n-3 | 0.051 | 0.058 |
|  | C20:4n-6 | 0.074 | 0.005* |
|  | C20:5n-3 | 0.135 | <0.001* |
|  | C22:5n-3 | 0.004 | 0.884 |
|  | C22:6n-3 | 0.237 | <0.001* |

To assess the potential of dietary intervention in increasing circulating FAs levels, we further estimated the correlations between the dietary FA intakes and their circulating concentrations. We screened a subset of the population that had data on both dietary FA intakes and its corresponding circulating concentration (N = 1398). Spearman rank correlation analysis was employed to quantify the correlations. FA indicates fatty acid.

^*^P value <0.0

**Table S9.** Association between VLSFAs and NfL with further adjustment for dietary FA intakes (N = 1398)

| VLSFAs | Range | Beta | 95% CI | P value |
| --- | --- | --- | --- | --- |
| Docosanoic acid (C22:0) |  |  |  |  |
|  | Overall | -0.105 | (-0.169, -0.041) | 0.001* |
|  | <Median | -0.217 | (-0.452, 0.018) | 0.071 |
|  | ≥Median | -0.024 | (-0.084, 0.036) | 0.432 |
| Tricosanoic acid (C23:0) |  |  |  |  |
|  | Overall | -0.237 | (-0.376, -0.097) | 0.001* |
|  | <Median | -0.594 | (-0.966, -0.222) | 0.002* |
|  | ≥Median | -0.168 | (-0.412, 0.076) | 0.178 |
| Lignoceric acid (C24:0) |  |  |  |  |
|  | Overall | -0.101 | (-0.173, -0.028) | 0.007* |
|  | <Median | -0.328 | (-0.535, -0.122) | 0.002* |
|  | ≥Median | -0.061 | (-0.194, 0.071) | 0.367 |

Linear regression models were employed to assess the association between circulating VLSFAs and serum NfL concentration in different ranges (overall/ < median/ ≥ median). The median value was 66.6μmol/L for C22:0, 28.5μmol/L for C23:0, and 56.4μmol/L for C24:0, respectively. All models were adjusted for covariates, including age, sex, race/ethnicity, BMI, educational attainment, PIR, marital status, smoking status, alcohol consumption, leisure-time PA, HEI-2015, FBG, triglyceride, serum creatinine. Also, models were additionally adjusted for dietary FA intakes (saturated, monounsaturated, and polyunsaturated). VLSFAs indicates very long-chain saturated fatty acids; NfL, neurofilament light chain; CI, confidence interval; others, refer to Table 1.

^*^P value <0.05
